# The choreography of the chemical defensome response to insecticide stress: insights into the *Anopheles stephensi* transcriptome using RNA-Seq

**DOI:** 10.1038/srep41312

**Published:** 2017-01-23

**Authors:** Leone De Marco, Davide Sassera, Sara Epis, Valentina Mastrantonio, Marco Ferrari, Irene Ricci, Francesco Comandatore, Claudio Bandi, Daniele Porretta, Sandra Urbanelli

**Affiliations:** 1 Department of Biology and Biotechnology, University of Pavia, Pavia, Italy; 2 School of Bioscience and Veterinary Medicine, University of Camerino, Camerino, Italy; 3 Department of Biosciences, University of Milan, Milan, Italy; 4 Department of Veterinary Medicine, University of Milan, Milan, Italy; 5 Department of Environmental Biology, Sapienza, University of Rome, Rome, Italy

## Abstract

Animals respond to chemical stress with an array of gene families and pathways termed “chemical defensome”. In arthropods, despite many defensome genes have been detected, how their activation is arranged during toxic exposure remains poorly understood. Here, we sequenced the transcriptome of *Anopheles stephensi* larvae exposed for six, 24 and 48 hours to the LD_50_ dose of the insecticide permethrin to monitor transcriptional changes of defensome genes across time. A total of 177 genes involved in insecticide defense were differentially expressed (DE) in at least one time-point, including genes encoding for Phase 0, I, II, III and antioxidant enzymes and for Heat Shock and Cuticular Proteins. Three major patterns emerged throughout time. First, most of DE genes were down-regulated at all time-points, suggesting a reallocation of energetic resources during insecticide stress. Second, single genes and clusters of genes turn off and on from six to 48 hours of treatment, showing a modulated response across time. Third, the number of up-regulated genes peaked at six hours and then decreased during exposure. Our results give a first picture of how defensome gene families respond against toxicants and provide a valuable resource for understanding how defensome genes work together during insecticide stress.

A major challenge for animals is to maintain homeostasis when exposed to chemical stressors, such as endogenous toxic chemicals or natural and synthetic xenobiotic compounds. These toxicants have likely acted as selective factors for the evolution of an array of gene families and pathways termed chemical defensome^1^, that allows an organism to sense, transform, and eliminate toxic chemicals. Comparative genomic analyses revealed the genetic redundancy and evolutionary conservation among metazoans of these gene families, that may constitute up to 2–3% of the total genome content^2^,^3^.

Central in the chemical defensome is the biotransformation system, which comprises genes encoding for several classes of proteins that modify the toxic compound making it harmless (Detoxifying Metabolic Enzymes, DMEs). Two main phases of this detoxification process have been recognized. Phase I is characterized by the oxidation, reduction, or hydrolysis of the toxic compounds by oxidative enzymes such as cytochromes P450 (CYPs), reductive enzymes such as aldo-ketoreductases (AKRs), or epoxide hidrolases (EHs) and by the activity of carboxylesterases enzymes (CCEs)^1^,^2^,^3^,^4^,^5^,^6^. Phase II metabolism involves conjugation, mostly of the already oxidized chemicals, with cellular glutathione, glucuronide, or other small hydrophilic molecules by transferase enzymes as glutathione S-tansferases (GSTs), sulfotransferases (SULT), UDP-glucuronosyltransferases (UGTs)^3^,^7^,^8^. Since the 1990s, the contribute of efflux pumps to detoxification, in concert with the biotransformation system, has emerged. Detoxifying efflux pumps are proteins located in the cellular membrane, belonging to the ATP-binding Cassette transporter family (ABC transporters). ABC transporters added two additional, essential steps to the defense mechanisms against xenobiotics (Phase 0 and Phase III)^3^,^9^. In Phase 0, ABC-transporters actively reduce the intracellular concentration of toxicants by preventing their entry into cells or by pumping them outside the cell once they entered, while in Phase III, they expel out of the cell the toxicants that were modified by detoxifying enzymes during Phases I and II. Finally, it is now generally acknowledged that the chemical defensome is comprehensive, along with the biotransformation system, of genes enconding for antioxidant enzymes that protect cells against reactive oxygen species (ROS) generated during biotransformation and of transcription factors that act as sensors for toxicants or cellular damage^3^.

The defense mechanism against natural and synthetic xenobiotics has long been investigated in arthropod species because of their great economic, environmental and medical-veterinary importance, as impollinators, agricultural pests, and vectors of human and animal diseases. Over the last decades, synergistic, enzymatic, genetic, and transcriptional studies have highlighted the involvement of metabolic detoxification in both resistance and defense to insecticides in a wide range of taxa^10^,^11^,^12^. Over-expression of genes encoding for proteins of the biotransformation system has been observed in insecticide resistant strains as well as transcriptional induction of defensome members has been observed in susceptible strains exposed to insecticides. More recently, whole transcriptome analyses, which allow to observe the turning on and off of thousands of genes in response to toxic compounds, are showing a role for previously overlooked gene families, such as genes encoding for Heat Shock Proteins (HSPs) and Cuticular Proteins (CPs)^13^,^14^,^15^.

Despite the increased focus on the components of the chemical defense, the sequence of the events that occur during toxic exposure remains poorly understood. Transcriptomic studies that have focused on single time-points showed that the exposure of individuals to toxicants induces up-regulation of several genes, while several others are down-regulated^16^,^17^,^18^,^19^,^20^,^21^. However, these are single snapshots of the defense response that do not allow us to know if and how the differentially expressed genes are modulated during toxicant exposure. On the other hand, some studies have investigated the expression profiles of subsets of genes at different time-points during individuals’ exposure to toxicants^14^,^22^,^23^,^24^,^25^,^26^. For example, in the mosquito *Culex quinquesfasciatus*, multiple P450 genes were found co-upregulated in resistant strains during a time course of 12, 24, 48 and 72 hours of permethrin exposure^24^,^25^. Likewise, in larvae of a susceptible strain of the mosquito *Anopheles stephensi* exposed to permethrin, it was analysed the expression pattern of six ABC transporter genes at seven time-points from 30 minutes to 48 hours after exposure. All of these genes were found differentially expressed compared to the untreated larvae at each time-point and showed a modulated transcriptional response across time, with the maximum up-regulation after six hours of exposure^22^. Similar patterns were also found for genes encoding for Cuticular Proteins in the mosquito *Culex pipiens pallens*^23^. These studies support the view that defensome genes likely operate continuously, turning off and on at different time-points. However, because these studies are based on a subset of defensome genes, just a partial picture of the whole defensome choreography has been so far provided.

In light of the above, we used RNA-Seq to massively detect differentially expressed genes in larvae of the main urban Asian malaria vector *An. stephensi* after exposure to permethrin, one of the most used synthetic insecticides. We exposed larvae of a susceptible strain of this mosquito (Liston) to the LD_50_ dose of permethrin and analysed the transcriptional response of the survived larvae (i.e. those that more efficiently defended themselves) after six, 24 and 48 hours of insecticide exposure. By using a high-throughput technique, i.e. RNA-Seq, with measurements taken at several time-points, we were able to obtain a more thorough picture of the chemical defensome, which allowed us to *i*) assess how many and which defensome genes were differentially expressed after insecticide exposure; *ii*) describe the transcriptional response across time of defensome gene families.

## Results

### Permethrin exposure

Third-instar larvae were exposed to 0.137 mg/L of permethrin^22^. Larval mortality after six, 24, and 48 hours of permethrin exposure was 33%, 45%, and 76% respectively. In control tests larval mortality was 2% after six- (C-6 h control-test), 2% after 24- (C-24 h control-test), and 1% after 48-hours of exposure (C-48 h control-test).

Three pools of fifty living larvae were collected from both control- and test-trays after six and 24 hours of permethrin exposure and stored for molecular analyses. Two pools of fifty larvae were collected from the 48 hours test-tray, because of the high larval mortality observed at this time-point.

Ten living larvae were collected from both test- and control-trays and used to assess the larval health status after exposure to permetrhin by analysing diving and feeding activities. The Shapiro-Wilk test showed normal distribution of the data (all tests *P* > 0.05). Diving rates observed were significantly lower (t-tests *P* < 0.05) in larvae exposed to permethrin than in untreated larvae at all the measured time-points. On the contrary, no significant differences in diving rates were observed between all control samples, as well as between treated samples at all time-points (t-tests *P > *0.05). Likewise, the time spent for feeding was lower in larvae exposed to permethrin than in control larvae at each time-point (t-tests *P* < 0.05), while no significant differences were detected between all control samples as well as between treated samples at six, 24 and 48 hours (all t-tests *P* > 0.05) (Supplementary Table S1).

### Sequencing

A total of 17 cDNA libraries were constructed and sequenced: 3 libraries for the three pools of larvae exposed to permethrin for six hours (T1–6 h, T2-6 h and T3-6 h) and 3 for the control pools (C1-6 h, C2-6 h and C3-6 h); 3 libraries for the three pools of larvae exposed to permethrin for 24 hours (T1-24 h, T2-24 h and T3-24 h) and 3 for the control pools (C1-24 h, C2-24 h and C3-24 h); 2 libraries for the two pools of larvae exposed to permethrin for 48 hours (T1-48 h, T2-48 h) and 3 for the control pools (C1-48 h, C2-48 h and C3-48 h) (Supplementary Table S2). High quality paired-end short reads obtained ranged from 8,075,568 (T3-6 h library) to 26,742,198 (T2-48 h library) with an average number of reads of 16,544,552 (Table 1; Supplementary Table S2).

### Mapping and Annotation

The percentage of reads mapping to the reference genome of *Anopheles stephensi* (AsteI2.2, Indian strain) ranged from 83.79% (T2-6 h library) to 89.09% (C1-24 h library) with an average mapping rate of 86.3% (Table 1 and Supplementary Table S2).

A total of 9,388 genes were found expressed. Annotation by BlastP search of *An. stephensi* genes against a custom mosquito protein database resulted in 8,033 proteins recognized with a significant e-value (*P* < 1e^−5^). Proteins annotated using a Gene Ontology (GO) approach were 7,599, with 1,981 unique GO terms (Table 1). Additionally, we characterized 6,748 proteins with EuKaryotic Orthologous Groups (KOG)^27^ and 4,792 with BlastKOALA (4,136 unique Kyoto Encyclopedia of Genes and Genomes orthologies) (KEGG)^28^. A total of 272 insecticide defense-related genes were found (Supplementary Table S3). Among them, 30 ABC transporter genes, belonging to all known ABC subfamilies but ABCH, were identified using a BlastP search and classified using a phylogenetic approach (5 ABCA, 4 ABCB, 8 ABCC, 2 ABCD, 1 ABCE, 3 ABCF and 7 ABCG) (Supplementary Fig. S1).

### Differential Expression analysis

#### Transcriptome changes during permethrin exposure

**Figure 1:**
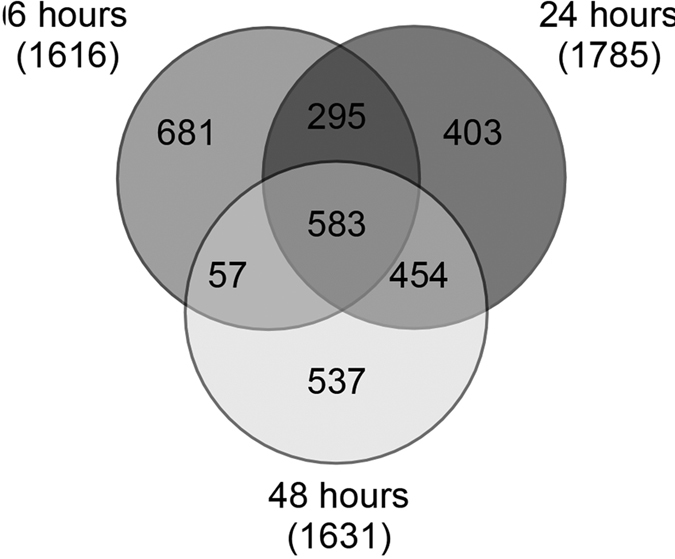
Venn diagram. Differentially expressed genes found in *Anopheles stephensi* in at least one time-point after permethrin exposure.

Thirty-two percent of the 9,388 expressed genes (2,986/9,388) were differentially expressed (DE) in at least one time-point over the 48 hours of permethrin exposure. The Venn diagram in Fig. 1 shows the number of shared and exclusive DE genes at each time-point. About 20% (583 genes) of the DE genes were expressed in response to permethrin over all of the 48 hours time-course, while 23% (681 genes) were differentially expressed exclusively at six hours after insecticide exposure, 13.5% (403 genes) were exclusive of 24 hours, and 18% (537 genes) exclusive of 48 hours after exposure. Some other genes were commonly expressed at six and 24 hours (9.8%), at 24 and 48 hours (15.2%), and at six and 48 hours (1.9%).

**Figure 2:**
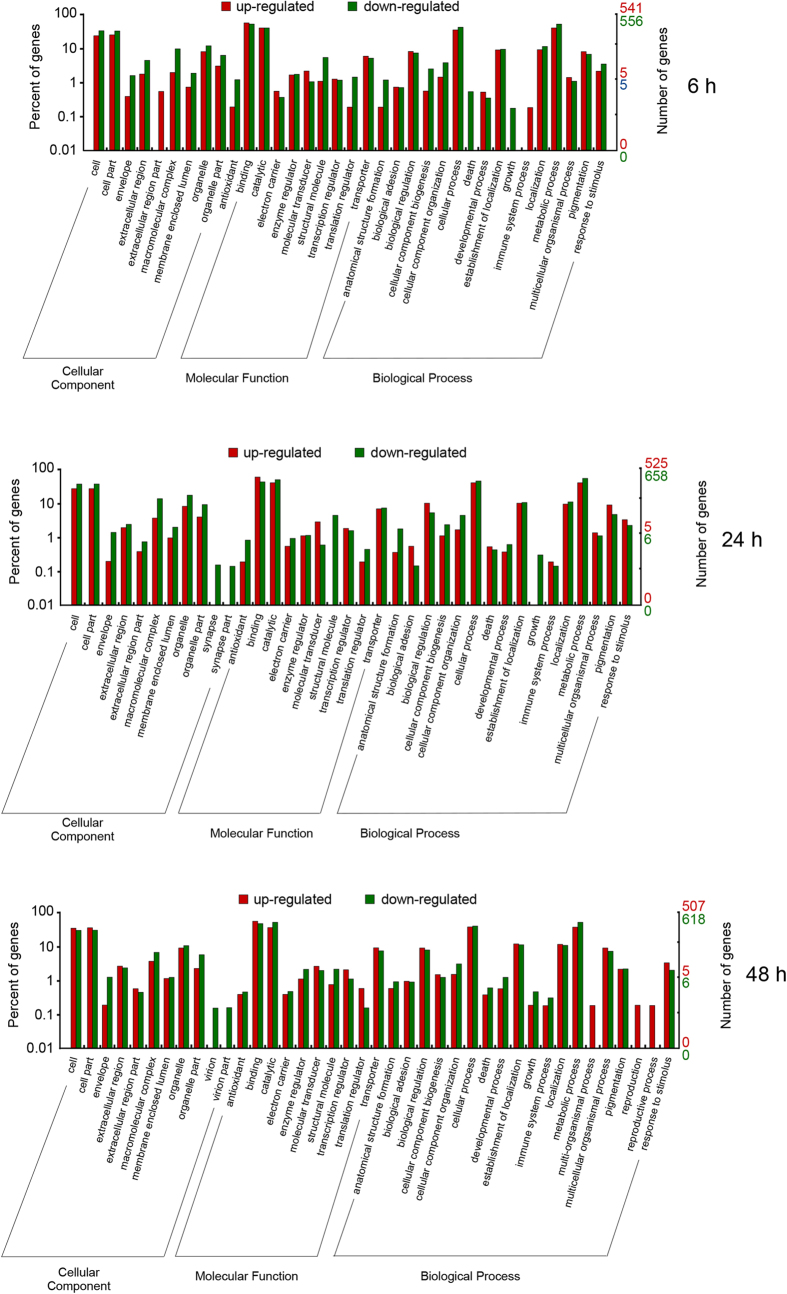
GO analysis by ontology categories. The fraction of genes classified in “Molecular function”, “Biological process” and “Cellular component” categories at six, 24 and 48 hours after permethrin exposure is shown.

Functional transcriptome changes after permethrin exposure were assessed by classifying gene functions using a GO analysis. In total, forty-two GO terms of the three GO categories (cellular component, molecular function and biological process) were assigned across the 48 hours of exposure (Fig. 2). Among them, 35 sub-categories were assigned at six hours after permethrin exposure (9 in cellular component; 10 in molecular function, and 16 in biological process). The major sub-categories within each category were cell and cell part (324 GO terms), binding (618 GO terms) and metabolic process (513 GO terms), respectively. After 24 hours of insecticide exposure, 37 sub-categories were attributed as follows: 11 in cellular component (cell and cell part were the major sub-categories with 391 GO terms); 10 in molecular function (binding was the major sub-category with 614 GO terms); 16 in biological process (metabolic process was the major sub-category, 580 GO terms). After 48 hours of insecticide exposure, 40 sub-categories were found: 11 in cellular component (364 GO terms in cell and cell part); 10 in molecular function (587 GO terms in binding); 19 in biological process (518 GO terms in metabolic process) (Fig. 2).

**Figure 3:**
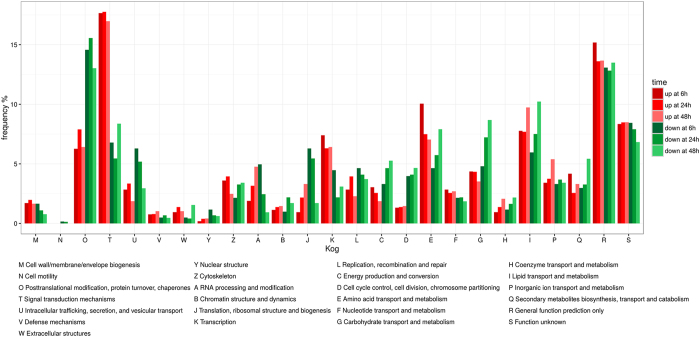
Clusters of Orthologous Groups function classification. Up- and down-regulated genes are shown for each functional category for six, 24 and 48 hours after permethrin exposure.

In total, 2,048 out of the 2,986 differentially expressed genes were annotated into 25 KOG categories (Fig. 3). Among them, the cluster “general function prediction” was the largest (159; 28.26%, 163; 26.43%, 153; 27.15% at six, 24 and 48 hours of permethrin exposure, respectively), followed by “signal transduction mechanisms” (134; 24.44%, 130; 23.21%, 136; 25.31% at six, 24 and 48 hours, respectively) which also showed the highest number of up-regulated genes at all time points (Fig. 3). The smallest group was “cell motility” (1; 0.17%, 1; 0.14%, and 0 at six, 24 and 48 hours, respectively) followed by “nuclear structure” (8; 1.35%, 7; 1.07%, 6; 1.03%). Finally, 1,396 differentially expressed genes were characterized with 1,193 KEGG orthologies. Within the pathways belonging to “xenobiotics biodegradation and metabolism”, we found 52 differentially expressed genes. This subset included 22 defensome genes encoding for Phase 0/III, I and II enzymes, as well as 30 further genes which could be involved in the response to insecticide (Supplementary Table S3 and Table S4).

#### Defensome expression changes during permethrin exposure

A total of 272 genes involved in insecticide defense were found and among them 177 were differentially expressed in at least one time-point (Supplementary Table S3 and S4). Sixty-six Phase I genes were detected and among them 67% (44/66) were differentially expressed over the 48 hours of permethrin exposure (25, 22 and 30 genes were differentially expressed after six, 24 and 48 hours of exposure, respectively) (Table 2). Up- and down-regulated genes at the different time-points are shown in the Venn diagrams in Supplementary Fig. S2. With respect to the up-regulated genes, three were found up-regulated at all time points analysed over the 48 hours time course, while seven were up-regulated exclusively at six hours after insecticide exposure, one exclusively at 24 h and three exclusively at 48 hours (Supplementary Table S3 and Fig. S2).

Thirty-five Phase II genes were found and 68.5% (24/35) were differentially expressed in at least one time-point over the 48 hours of permethrin exposure (four, 20 and 23 genes after six, 24 and 48 hours of exposure, respectively) (Table 2).

Nineteen Phase 0/III genes encoding for ABC transporters, belonging to the ABCB, ABCC and ABCG subfamilies, were found and 53% of them (10/19) were differentially expressed in at least one time-point (five, eight and seven genes after six, 24 and 48 hours, respectively) (Table 2). Two ABC transporter genes were up-regulated at all time-points analysed over the 48 hours time course, while one gene was found up-regulated exclusively at six hours and one after 48 hours of exposure (Table 2; Supplementary Fig. S2).

In addition to Phase I, II and 0/III genes, other genes related to the defensome were found differentially expressed. Among these, genes enconding for antioxidant enzymes (one catalase and five superoxide dismutases encoding genes), transcription factors that act as sensors of toxicants or cellular damage, such as the aryl hydrocarbon receptor (Ahr), nuclear factors (NRs), the mitogen-activated protein kinase (MAPK) signaling pathways and the nuclear factor erythroid (Nfr2) have been detected (Supplementary Table S3). Finally, other xenobiotics defense-related genes and genes known to be involved in the general stress response were found differentially expressed, such as the genes encoding for Heat Shock Proteins (10 genes) and Cuticular Proteins (68 genes) (Table 2, Supplementary Table S3 and Fig. S2).

**Figure 4:**
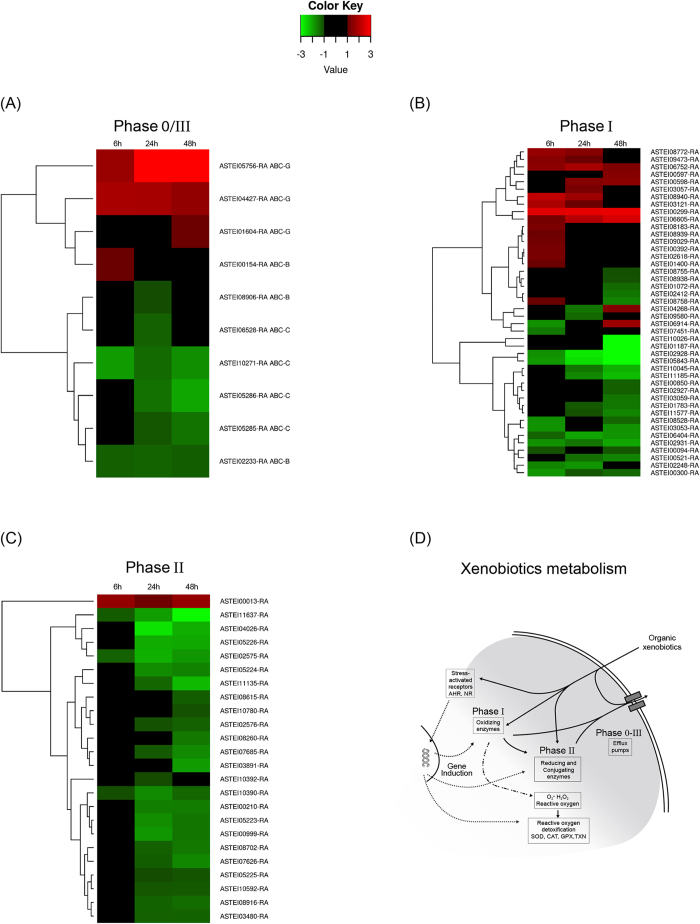
Differentially expressed genes in mosquito larvae exposed to permethrin at six, 24 and 48 h. Hierarchical clustering analysis based on their log fold-change. Genes not differentially expressed in any time-point were excluded from the analysis. For each gene, the ID is also indicated. (**A**) Phase 0/III enzymes: ABC transporters; (**B**) Phase I enzymes: Cytochrome P450 and Carboxylesterases; (**C**) Phase II enzymes: Glutathione S-transferases and UDP-glucuronosyltransferases. (**D**) Graphical representation of the chemical defensome. In the figure, the detoxifying metabolic enzymes and the pathways involved are shown. The figure was traced and modified from^1^, using the software Canvas 15 (ACD systems http://www.acdsee.com/de/products/canvas-15).

The temporal expression profiles of Phase I, II, and 0/III genes were further investigated by clustering the members of each sub-group based on their log fold-change (Fig. 4; Supplementary Fig. S3). Phase 0/III genes clustered in two main groups. The first one, made up of all up-regulated genes, was composed mostly of ABCG transporters (3 genes) and one ABCB transporter; in the second group, containing all down-regulated genes, only ABC transporters belonging to the ABCB and ABCC sub-families were found (Fig. 4A).

The Phase I genes clustered into two main groups and several sub-groups containing both CYPs and CCEs genes. Some of them contained only up-regulated genes after at least one time-point, some others contained both up- and down-regulated genes or only down-regulated genes (Fig. 4B). All Phase II genes, with the exception of one UGT gene (ASTEI00013-RA) clustered into several sub-groups containing both GSTs and UGTs genes that were down-regulated mostly after 24 and 48 hours of exposure (Fig. 4C).

Cluster analysis was also performed for differentially expressed genes encoding for Cuticular Proteins (CPs), resulting in two main groups and several sub-groups. Cluster I contained all down-regulated genes but one, that was up-regulated at six hours of exposure. Cluster II was subdivided into further sub-groups. The IIa1 contained only genes up-regulated after 48 hours of exposure, while the IIa2 group contained only genes up-regulated after six hours. The group IIb contained only down-regulated genes after six hours of permethrin exposure (Supplementary Fig. S4).

### Reverse Transcription Quantitative Real-Time PCR (RT-qPCR) validation

Eight transcripts, detected as differentially expressed between treated and untreated mosquito larvae by RNA-Seq at different time-points were used for validation by RT-qPCR analysis. All genes showed a concordant pattern of up- or down-regulation between RNA-Seq and RT-qPCR data (Supplementary Fig. S5).

## Discussion

### Defensome genes in *Anopheles stephensi* larvae

Permethrin, like all pyrethroid insecticides, is a neurotoxic compounds whose main target is the voltage gated sodium channel^29^. In this study, we exposed larvae of a susceptible strain of the mosquito *Anopheles stephensi* to permethrin insecticide and analysed the transcriptional response after six, 24, and 48 hours of exposure to investigate the defensome response to insecticide stress across time. The involvement of detoxification complex enzymes in both resistance and defense to pyrethroids has been documented in several arthropod species, including mosquitoes^6^,^11^,^12^. Among them, cytochromes P450 (CYPs), carboxylesterases (CCEs), glutathione S-transferases (GSTs), UDP-glucuronosyl transferases (UGTs) enzymes, as well as ABC transporters were found differentially expressed between resistant and susceptible strains to pyrethroids^16^,^17^,^18^,^30^,^31^. Likewise, induced up-regulation of detoxifying genes was found in resistant individuals exposed to pyrethroids, such as in the mosquito *Anopheles gambiae*^30^, the cattle tick *Rhipicephalus (Boophilus) microplus*^32^ or the mosquito *Culex quinquefasciatus*^24^,^25^, as well as in susceptible individuals, such as in *Panonychus citri*^19^, *Liposcelis bostrychophila*^20^, and *Melita plumulosa*^21^.

Concordantly with the above studies, we annotated 272 genes in the genome of *An. stephensi* involved in insecticide defense and found extensive transcriptional changes between larvae exposed and not-exposed to permethrin. Sixty-six Phase I genes were found, including several CYPs and CCEs, the main enzymatic complexes acting in this phase, and 29% of them were up-regulated in at least one time-point over the 48 hours of permethrin exposure, which supports a major role of these detoxifing enzymes in defense against permethrin. Phase II genes found included GSTs and UGTs (22 and 13 genes, respectively) and, among them, one UGT gene was up-regulated, while all GSTs were down-regulated in treated larvae. Down-regulation or no-differential expression of GSTs encoding genes after pyrethroid exposure has been documented in other insect species. For example, in third instar-nymphs of the rice planthoppers *Sogatella furcifera* exposed to the LD_20_ concentration of beta-cypermethrin, three and six out of the nine GSTs genes found were detected no differentially expressed and down-regulated at 24 h, respectively^26^. Likewise, in the booklouse *Liposcelis bostrychophila* exposed to the LD_20_ dose of deltamethrin, none out of 31 GST genes found were differentially expressed between treated and untreated individuals^20^. GSTs have been suggested to catalyse different detoxification reactions, such as conjugation under exposure to organophosphates insecticides, or conjugation and dehydrochlorination in response to organochlorines^8^. Against pyrethroids, they would act by reducing peroxidative damage through detoxification of lipid peroxidation products. This indirect detoxifying role could account for their absent up-regulation in the time-window analysed in our study, concordantly with the scarce up-regulation observed in other genes encoding for antioxidant enzymes (one superoxide dismutase antioxidant gene) (Supplementary Table S3). In this context, it could be hypothesized that the constitutive activity of GSTs and antioxidant enzymes is enough to attenuate the accumulation of ROS generated by Phase I enzymes or that antioxidant transcriptional response could be activated in time-points out of the time window analysed under our experimental conditions^14^. Nineteen Phase 0/III genes were found, comprising members of all ABC-transporters sub-families known to be involved in insecticide detoxification^9^, with four of them (21%) detected as up-regulated in at least one time point of permethrin exposure. These results are concordant with our previous studies on induction of ABC transporters in *An. stephensi* by permethrin^22^,^33^ and, more in general, with the even more numerous evidences for ABC transporters involvement in arthropod defense against insecticides^9^,^22^,^33^,^34^,^35^,^36^,^37^,^38^,^39^.

It is interesting to note that some genes that we found differentially expressed in *An. stephensi* larvae had already been shown to be involved in pyrethroid resistance in mosquitoes, although caution should be taken when comparing different species, strains (resistant and susceptible) and results from different experimental designs (e.g. induced vs constitutive expression profile analyses). For example, the CYP352C2 gene (ASTEI00597-RA) that we found up-regulated, is known to be constitutively up-regulated in an *Anopheles arabiensis* strain resistant to deltamethrin^40^. On the other hand, some other genes that we found down-regulated or not differentially expressed, were found constitutively up-regulated in a pyretroid-resistant strain of *An. stephensi*^41^ (i.e. the GSTs ASTEI05223-RA, ASTEI09484-RA and ASTEI10780-RA; the CYP450, ASTEI02412-RA, and the CCEs, ASTEI08528-RA encoding genes) (Supplementary Table S3). The investigation of factors underlying the heterogeneity of defensome response between species or resistant and susceptible strains of the same species remains an unanswered and exciting topic for future researches^38^.

Defensome genes include transcription factors that act as sensors of toxicants or cellular damage. We annotated and detected 38 genes encoding for transcription factors being expressed, including the aryl hydrocarbon receptor (Ahr), nuclear factors (NRs), mitogen-activated protein kinase (MAPK) and the nuclear factor erythroid (Nfr2). Among them, we detected up-regulation for one gene encoding for the Aryl hydrocarbon receptor interacting protein after 24 hours of exposure (ASTEI04432-RA) (Supplementary Table S3), a component of the Ahr pathways that regulate the Phase I genes expression, one gene encoding for a MAP kinase after 24 and 48 hours (ASTEI06997-RA), and one encoding for a Nrf2 after 24 hours (ASTEI05762-RA), which are both involved in the induction of genes encoding for antioxidant enzymes (Supplementary Tables S3)^3^,^42^,^43^. These results support the occurrence of all these signaling pathways in *An. stephensi* and their putative role in insecticide defense [see ref. 44]. Notably, among the DE genes, ten Cuticular Proteins were up-regulated in treated larvae, which further support the recent data showing their important role in insecticide defense and resistance^23^,^45^,^46^.

### Defensome transcriptional changes during permethrin exposure

Three major patterns can be observed in the transcriptional changes of defensome genes during permethrin exposure.

First, the greatest fraction of defensome genes differentially expressed after six, 24 and 48 hours of treatment was down-regulated (Table 2, Fig. 3, Supplementary Table S3). This pattern is particularly evident in Phase II genes, where all but one gene were down-regulated, but it can be also observed in Phase I and Phase 0/III genes, as well as in the genes encoding for HSPs (70% down-regulated), and CPs (65% down-regulated). Furthermore, this pattern can be observed at both all and single time-point analysed (Table 2). Permethrin exposure can be extremely costly from an energetic point of view. The target site of permethrin is the GABA receptor and the effect of its hyperactivation by permethrin leads to energy loss. Likewise, the defense response is energetically costly as a consequence of the transcriptional induction, protein synthesis, and detoxification activity and could lead to drastic metabolic changes in individuals exposed to insecticides and a reallocation of energy resources^47^,^48^. For example, in *Apis mellifera,* up-regulation of detoxifying genes by the neonicotinoid insecticide imidacloprid was coupled with down-regulation of genes associated with glycolisis and development^49^. Coherently with this, as suggested by the GO and KOG analyses, we found drastic transcriptional changes in all categories and sub-categories throughout all times of exposure. For example, genes belonging to the GO growth sub-category were down-regulated after six and 24 hours (Fig. 2). Furthermore, we found a reduction of larval diving and feeding activities in exposed larvae at all time points compared to controls. These changes could therefore be part of a general reallocation of energetic resources which could account as well for the fraction of down-regulated defensome genes.

Secondly, when we look at the transcriptome changes during insecticide exposure, a modulated response of defensome genes across time can be observed at multiple levels. The first one was the gene level. The expression pattern of differentially expressed genes ranged from down- to up-regulation at all time-points, encompassing all possible combinations of up-/down-regulation at each time-point. For example, the ASTEI06914 gene (Phase I, CYP18A1) was down-regulated, not differentially expressed, and up-regulated at six, 24 and 48 hours, respectively. The opposite expression pattern was found in the ASTEI08758 gene (Phase I, CYP4H14). These results confirm and extend the findings of those studies that analysed across time the expression profile of genes involved in insecticide defense, such as the CYPs^24^,^25^, the ABC transporters^22^, or the CPs^23^. A further level of modulation of defensome response can be observed at the gene family level. Within each family, some genes were up- or down-regulated at all time-points, while others were differentially expressed only in one or two time-points (Fig. 3). Finally, a modulation of the transcriptional response from six to 48 hours of permethrin exposure can be observed at the whole defensome level. Groups of genes with similar expression patterns can be also observed when we consider all differentially expressed defensome genes. The gene groups and sub-groups found at each time-point include genes belonging to different gene families. Interestingly, it can also be observed that the response to insecticide is not due to high up-regulation of few genes, but rather to the up-regulation of several genes showing similar values of expression, suggesting the induction of multiple defensome members that overlap across time.

Finally, the number of up-regulated genes peaked at six hours and then decreased during exposure. At six hours of exposure 17% of defensome genes were up-regulated, while at 24 and 48 h of exposure the 10% and 12% of up-regulated genes were observed. The deleterious effects of permethrin as well as the energetic cost of detoxification could gradually weaken the larvae, reducing their defense response across time. At least three sets of evidences in our data led to consider this hypothesis unlikely: *i*) the results of larval activity tests showed no significant differences between the larvae exposed to permethrin for six, 24 and 48 hours, suggesting no differences in the health status of the surviving larvae between the time-points analysed (Supplementary Table S1); *ii*) the overall number of up-regulated genes decreased across time, but a coherent reduction according to their function can be observed between the detoxifying metabolic enzymes. For example, in Phase 0/III genes, slight differences were observed from six to 48 hours, which is consistent with the role of the ABC transporters in defense response: on one hand they would act as the first line of cellular defense by pumping out from the cell the un-modified insecticide molecules; on the other hand, they act expelling the insecticides modified by Phase I and II enzymes at the end of the biotransformation process. Likewise, Phase I genes were mostly up-regulated after six hours of exposure and then their expression decreased at 24 and 48 hours, which is consistent with their role in insecticide metabolism (Table 2, Fig. 4D). *iii*) As discussed above, the transcriptional response is modulated at different levels: some genes and groups of genes turn off from six to 48 hours of treatment, but some others switch on across time, which can hardly be explained by a gradual larval weakening. On the contrary, these findings support the view that the observed temporal changes are due to a modulated defensome response across time, that would be stronger in the early stages of exposure and then be reduced with the ongoing of the detoxification process. It might be premature to speculate on the generality of the above patterns as the exposure conditions (i.e. time of exposure and toxicant dose) can greatly affect the defensome response, as well as it can differ among species, strains, developmental stages or sexes^14^,^38^. However, this study is the first attempt to give a picture of the temporal defensome expression dynamics during insecticide stress and provides a valuable resource for understanding how defensome genes work together.

## Conclusions

Ecological transcriptomics has proved to be a powerful tool to investigate how organisms cope with xenobiotic stressors. Detoxification enzymes play important roles in the metabolism of insecticides in insects and they can rapidly increase their activity in response to chemical stress, a phenomenon known as enzyme induction. Transcriptional studies allowed to highlight that the induction of detoxification enzymes involves the synthesis of new enzymes rather than the activation of preexisting enzymes or a block in the rate of degradation^4^. Indeed, genes encoding for proteins of the biotransformation system have been observed constitutively over-expressed in insecticide resistant strains while transcriptional induction of defensome members has been observed in susceptible strains exposed to insecticides^4^,^50^. More recently, whole transcriptome analyses greatly improved the detection of the genes involved in defense against insecticides. The analysis of changes in gene expression across time during chemical stress is the needed further step to move from gene inventories to the unraveling of gene interactions and regulation pathways. In this context, the results obtained in this study provide a first dynamic picture of defensome response to insecticides and a framework for future studies.

## Materials and Methods

### Mosquito samples and permethrin exposure

The mosquito larvae used in this study come from an *An. stephensi* (Liston) insecticide-susceptible strain maintained in the insectary of the University of Camerino, Italy. Newly hatched first instar larvae were maintained at 29 °C temperature, 85–90% relative humidity, 12:12 L-D photoperiod in 21 × 25 × 9 cm plastic trays filled with 2 liters of spring water and daily fed with fish food (Tetra, Melle, Germany)^33^. All experiments were conducted on *An. stephensi* third instar larvae.

In previous studies using the same *An. stephensi* strain, we analysed the transcriptional response of genes encoding for ABC transporters after 30 min., one, two, four, six, 24 and 48 hours of exposure to the LD_50_ dose of permethrin (0.137 mg/L). Significant gene up-regulation was detected mainly after six, 24 and 48 hours of exposure^22^,^33^. On the basis of these results, we treated the larvae with 0.137 mg/L of permethrin (PESTANAL^®^, C_21_H_20_Cl_2_O_3_, Sigma-Aldrich S.r.l., Milan, Italy) and analysed transcriptome changes by RNA-Seq after six, 24 and 48 hours of exposure.

Six experimental plastic trays (21 × 25 × 9 cm) filled with two liters of spring water and containing 500 larvae were set up: three trays for larval exposure tests (T-6 h, T-24 h, T-48 h, respectively) (0.137 mg/L permethrin), and three trays for the control tests without permethrin (C-6 h, C-24 h and C-48 h, respectively). After six hours of exposure, larval mortality was measured in test- and control-trays (T-6 h and C-6 h trays, respectively). Then, three pools of fifty living larvae were collected from the T-6 h test-tray (T1–6 h, T2-6 h and T3-6 h) and three from the C-6 h control-tray (C1-6 h, C2-6 h and C3-6 h), placed in RNAlater and stored at −80 °C for molecular analyses.

Ten additional larvae were collected from both test- and control-trays and used to assess larval health status after exposure to permetrhin. For this purpose, diving and feeding activities of larvae were analysed. Diving is a common larval behavior that consists of bottom-up movements in the water column of breeding sites^51^,^52^. Single larvae were placed into 250 ml plastic glasses filled with 100 ml of the same water of the experimental tray (water + permethrin). After 1 minute of larval naturalization, we registered the number of divings within 5 minutes of observations. We defined feeding behavior of larvae as three activities: movement to the food source, capture of food particles, and mouthpart movements. It was assessed by registering the time that each larva spent feeding within 5 minutes of each observation. Single larvae were placed into 250 ml plastic glasses filled with 100 ml of the same water of the experimental tray as above and containing 0.3 mg of fish food. The time spent feeding was then registered. Shapiro-Wilk test was performed to check normality of the data^53^, then the Student’s t-test was used for testing the differences between treated and control larvae and between the larvae exposed to permethrin at different time-points. All analyses were performed using the software R 3.0.2^54^.

The same procedures of permethrin exposure and activity assessment described above were followed using larvae exposed to permethrin for 24 (T-24 h tray) and 48 hours (T-48 h tray) and their respective controls (C-24 h and C-48 trays).

### RNA isolation, cDNA library construction and Sequencing

RNA was extracted from each pool of larvae stored in RNAlater with the RNAeasy Mini Kit (Qiagen, Hilden, Germany) following the manufacturer’s instructions. cDNA libraries preparation and sequencing were performed by Polo GGB, Perugia, Italy (http://www.pologgb.com/) in one run of 2 × 150 paired-end reads on a HiSeq-2500 platform (Illumina). Reads are available in the EBI Short Read Archive (Sample accession: ERS1203181- ERS1203197).

### Reads mapping and Annotation

After an assessment of the reads quality using FastQC^55^, high quality paired-end reads were mapped to the reference genome of *An. stephensi* retrieved from VectorBase (AsteI 2.2, Indian strain)^56^ using Bowtie 2^57^.

Gene sequences of the reference genome of *An. stephensi* were annotated using several approaches. Firstly, a similarity-based approach was used by creating a custom mosquito database of annotated proteins. Protein fasta files from five mosquito species (*Aedes aegypti, An. darlingi, An. gambiae, An. sinensis,* and *Culex quinquefasciatus*) were retrieved from VectorBase and unannotated sequences were removed. A BlastP^58^ search of *An. stephensi* proteins against this database was performed with cutoff e-value of 1E-05. Secondly, GO terms and putative domains were assigned to *An. stephensi* sequences using InterproScan5^59^.

Additionally, *An. stephensi* proteins were further characterized using the KOG and KEGG databases. Sequences were classified in one of the 25 KOG functional categories using Blast with a cutoff e-value of 1E-05. KEGG mapping was performed using the BlastKOALA webserver, while KEGG Mapper was used to reconstruct individual pathways. In addition, we exploited the already published annotation from the microarray study on *An. stephensi*^41^ to validate or expand our defensome annotation. Genes encoding for detoxifying families were retrieved from the microarray chip and identified in our reference genome with a double best hit blastn search. Finally, for a more confident assignment of ABC transporters to ABC sub-families, we mined the genome of *An. stephensi* for putative transporters. A comprehensive custom dataset was created by retrieving all ABC transporter sequences in Uniprot using the taxonomic filter Metazoa. *An. stephensi* ABC transporters were identified with a BlastP search against this database. Successively, putative *An. stephensi* ABC transporters were classified into families with a phylogenetic approach by creating a multi-alignment of all ABC transporter sequences, putative and Uniprot annotated, with Cobalt^60^ and using the result as input for RaxML^61^ with 1000 bootstrap replicates.

### Differential Expression analysis

Raw counts for each gene and each sample were extracted from SAM alignments using samtools^62^ and htseq-count^63^. A table of raw counts was used as input for DESeq2^64^ for the normalization step and differential expression analysis. Samples were analyzed separately following the DESeq2 software documentation, as they are distinct pools of different individuals randomly sampled (quasi replicates). Pairwise comparisons were made between controls and treated samples at each time-point. At any given time point, a gene was considered differentially expressed if its adjusted p-value (Benjamini-Hochberg adjustment) was less than 0.05 and its absolute log2 fold change was greater than 1. GO terms belonging to up- and down-regulated genes at each time-point were visualized using WeGO^65^. Similarly, the KOG characterization of differentially expressed genes was processed using R and visualized using ggplot2^66^. Finally, the expression profiles of defensome genes and Cuticular Proteins were investigated by clustering the members of each sub-group based on their log fold-change using gplots^67^.

### Reverse Transcription Quantitative Real-Time PCR (RT-qPCR) validation

The gene expression profile of eight differentially expressed genes obtained by RNA-Seq was validated by reverse transcription quantitative PCR (RT-qPCR). We chose at least one member of the detoxifying enzymatic families that we were interested in and that we observed being differentially expressed after insecticide exposure. Genes showing up- and/or down-regulation have been selected to validate differential expression in both directions. cDNAs were synthesized starting from 200 ng of total RNA using a QuantiTect Reverse Transcription Kit (Qiagen). The cDNA was used as template for RT-qPCRs using the primer pairs reported in Supplementary Table S4, derived from the sequences identified in the transcriptome. The amplification fragments obtained using standard PCR conditions and cycles were sequenced in order to confirm the specificity of the amplifications^68^. Quantitative RT-PCRs of target genes were performed using a CFX Connect Real-Time PCR Detection System (Bio-Rad, Hercules, CA, USA) with SYBR Green supermix (Bio-Rad), following the conditions reported in ref. 22.

## Additional Information

**How to cite this article**: De Marco, L. *et al*. The choreography of the chemical defensome response to insecticide stress: insights into the *Anopheles stephensi* transcriptome using RNA-Seq. *Sci. Rep.* **7**, 41312; doi: 10.1038/srep41312 (2017).

**Publisher's note:** Springer Nature remains neutral with regard to jurisdictional claims in published maps and institutional affiliations.

## Supplementary Material

Supplementary Information

## Figures and Tables

**Table 1:** Summary of *Anopheles stephensi* cDNA sequencing, mapping and annotation.

Description	Value
Average number of reads	16,544,552
Average mapping rate - %	86.30
Expressed genes	9,388
Annotated genes – BLAST p-value < 1^e−5^	8,033
Annotated genes – GO	7,599
Total unique GO terms	1,981
Annotated genes KOG	6,748
Annotates genes BlastKoala	4,792
Total unique KO	4,136

GO: gene onthology; KOG: EuKaryotic Orthologous Groups; KO: KEGG orthology.

**Table 2:** Defensome genes in larvae of *Anopheles stephensi.*

	N_tot_	N_DE_	N_No-DE_	6 h			24 h			48 h		
				Up	Down	No-DE	Up	Down	No-DE	Up	Down	No-DE
**Phase I**	**66**	**44**	**22**	**14**	**11**	**41**	**9**	**13**	**44**	**7**	**23**	**36**
Cytochromes P450 (CYPs)	44	33	11	9	9	26	6	9	29	5	18	21
Epoxide hydrolase (EHs)	1	0	1	0	0	1	0	0	1	0	0	1
Aldo-keto-reductase (AKRs)	1	0	1	0	0	1	0	0	1	0	0	1
Carboxylesterases (CCEs)	20	11	9	5	2	13	3	4	13	2	5	13
**Phase II**	**35**	**24**	**11**	**1**	**3**	**31**	**1**	**19**	**15**	**1**	**22**	**12**
Glutathione S-tansferases (GSTs)	22	14	8	0	2	20	0	13	9	0	14	8
UDP-glucuronosyltransferases (UGTs)	13	10	3	1	1	11	1	6	6	1	8	4
**Phase 0/III**	**19**	**10**	**9**	**3**	**2**	**14**	**2**	**6**	**11**	**3**	**4**	**12**
**Antioxidant Enzymes**	**6**	**2**	**4**	**1**	**0**	**5**	**1**	**1**	**4**	**1**	**1**	**4**
Catalases (CATs)	1	0	1	0	0	1	0	0	1	0	0	1
Superoxide dismutases (SODs)	5	2	3	1	0	4	1	1	3	1	1	3
**Heat Shock Proteins (HPs)**	**10**	**7**	**3**	**0**	**7**	**3**	**0**	**6**	**4**	**0**	**5**	**5**
**Cuticular Proteins (CPs)**	**68**	**54**	**14**	**5**	**33**	**30**	**0**	**3**	**65**	**5**	**11**	**52**

N_tot_: total number of defensome genes found, genes differentially expressed (DE) in at least one time point after permethrin exposure (N_DE_) and no differentially expressed in any time-point (N_NO-DE_). Up, Down and No-DE: genes up-, down-regulated and no differentially expressed with respect to controls after six, 24 and 48 hours of exposure to permethrin.
