# Supplementary material for: The choreography of the chemical defensome response to insecticide stress: insights into the Anopheles stephensi transcriptome using RNA-Seq
Source: Sci Rep. 2017 Jan 23;7:41312. doi: 10.1038/srep41312 (PMC5256098; doi:10.1038/srep41312)

The choreography of the chemical defensome response to insecticide stress: insights into the *Anopheles stephensi* transcriptome using RNA-Seq

Leone De Marco[1,2], Davide Sassera[1], Sara Epis[3,4], Valentina Mastrantonio[5], Marco Ferrari[3], Irene Ricci[2], Francesco Comandatore[3,4], Claudio Bandi[3], Daniele Porretta[5*] and Sandra Urbanelli[5]

[1]Department of Biology and Biotechnology, University of Pavia, Pavia, Italy;

[2]School of Bioscience and Veterinary Medicine, University of Camerino, Camerino, Italy.

[3]Department of Biosciences, University of Milan, Milan, Italy.

[4] Department of Veterinary Medicine, University of Milan, Milan, Italy.

[5] Department of Environmental Biology, Sapienza, University of Rome, Rome, Italy.

Table S1. Analysis of diving and feeding activities in *Anopheles stephensi* larvae. Diving is expressed as mean number of diving/larva within 5 minutes of observations. Feeding is expressed as the mean time of feeding/larva within 5 minutes of observations. Standard deviations are shown in brackets. Ten larvae were used in each treatment. Tests and controls refer to *An. stephensi* larvae exposed to permethrin for six, 24 and 48 hours. Letters refer to Student-t test significance: equal letters refer to no significant differences (P > 0.05); different letters, refer to significant differences (P < 0.05).

	**6 hours**		**24 hours**		**48 hours**	
	**Tests**	**Controls**	**Tests**	**Controls**	**Tests**	**Controls**
**Diving (n/larva)**	0.2^{a} (0.45)	1.5^{b}(0.58)	0.067^{a} (0.26)	2.06^{b} (1.24)	0.33^{a} (0.49)	1.3^{b}(0.78)
**Feeding (sec./larva)**	14.2^{A} (19.47)	66^{B} (36.26)	5.67^{A} (9.82)	150.3^{B} (99.53)	10.93^{A} (15.14)	115.56^{B} (56.864)

Table S2. Overview of *Anopheles stephensi* cDNA libraries sequenced. The accession summaries for each cDNA library are also shown. All libraries belong to the project classified in EBI Short Read Archive as PRJEB14386 (ERP016027).

Sample	Condition	Time	Number of reads	Reads mapped (%)	Sample accession	Secondary accession	Sample unique name
T1-6h	Permethrin treatment	6h	8,426,192	84.59	ERS1203190	SAMEA4032080	Aste_permethrin1_6
T2-6h	Permethrin treatment	6h	12,427,928	83.79	ERS1203191	SAMEA4032081	Aste_permethrin2_6
T3-6h	Permethrin treatment	6h	8,075,568	84.21	ERS1203192	SAMEA4032082	Aste_permethrin3_6
C1-6h	control	6h	20,424,530	87.25	ERS1203181	SAMEA4032071	Aste_control1_6
C2-6h	control	6h	19,186,242	87.46	ERS1203182	SAMEA4032072	Aste_control2_6
C3-6h	control	6h	19,525,044	87.24	ERS1203183	SAMEA4032073	Aste_control3_6
T1-24h	Permethrin treatment	24h	12,169,006	86.74	ERS1203193	SAMEA4032083	Aste_permethrin1_24
T2-24h	Permethrin treatment	24h	17,871,396	86.42	ERS1203194	SAMEA4032084	Aste_permethrin2_24
T3-24h	Permethrin treatment	24h	12,079,144	85.13	ERS1203195	SAMEA4032085	Aste_permethrin3_24
C1-24h	control	24h	14,718,886	89.09	ERS1203184	SAMEA4032074	Aste_control1_24
C2-24h	control	24h	11,084,542	84.5	ERS1203185	SAMEA4032075	Aste_control2_24
C3-24h	control	24h	24,048,844	86.87	ERS1203186	SAMEA4032076	Aste_control3_24
T1-48h	Permethrin treatment	48h	22,551,016	87.11	ERS1203196	SAMEA4032086	Aste_permethrin1_48
T2-48h	Permethrin treatment	48h	26,742,198	86.39	ERS1203197	SAMEA4032087	Aste_permethrin2_48
C1-48h	control	48h	20,156,386	87.62	ERS1203187	SAMEA4032077	Aste_control1_48
C2-48h	control	48h	18,498,212	86.53	ERS1203188	SAMEA4032078	Aste_control2_48
C3-48h	control	48h	13,272,258	86.19	ERS1203189	SAMEA4032079	Aste_control3_48

Table S3. List of defensome genes and their expression trend after six, 24 and 48 hours of permethrin exposure. Expression trend at six, 24 and 48 hours time points is encoded as: -1, down-regulated ; 0 no differential expression; 1, up-regulated (i.e. -1,-1,-1 means that the gene is down-regulated after six,24 and 48 hours of exposure). Genes encoding for Heat shock Proteins and Cuticular Proteins are also shown.

An. stephensi ID	Expression Trend	6 hours		24 hours		48 hours		Mosquito ID	Species	Annotation	e-value	KOG ID	KEGG ID
		log_2FC	*P*	log_2FC	*P*	log_2FC	*P*						
**Phase 0/III**													
ASTEI02233-RA	-1,-1,-1	-1.234	0.000	-1.300	0.007	-1.218	0.000	AGAP005639-PA	*An. gambiae*	ATP-binding cassette, subfamily B (MDR/TAP), member 1	0	KOG0055	K05658
ASTEI10271-RA	-1,-1,-1	-1.897	0.000	-1.353	0.000	-1.763	0.000	AGAP006427-PA	*An .gambiae*	ATP-binding cassette, sub-family C (CFTR/MRP), member 4	4E-102	KOG0054	NA
ASTEI05285-RA	0,-1,-1	-0.651	0.000	-1.136	0.000	-1.448	0.000	AGAP008436-PA	*An. gambiae*	ATP-binding cassette, sub-family C (CFTR/MRP), member 11	0	KOG0054	NA
ASTEI05286-RA	0,-1,-1	-0.256	0.027	-1.435	0.000	-2.029	0.000	AAEL005043-PA	*Ae. aegypti*	ATP-dependent bile acid permease	0	KOG0054	K05665
ASTEI08906-RA	0,-1,0	-0.512	0.020	-1.040	0.000	-0.374	0.144	AGAP006273-PA	*An. gambiae*	ATP-binding cassette, subfamily B (MDR/TAP), member 8	0	KOG0058	K056559
ASTEI06528-RA	0.-1,0	-0.306	0.104	-1.255	2.20e-13	-0.694	0.0003	ASTEI06528-RA	*Ae. aegypti*	Multidrug resistance protein 2 (ATP-binding cassette protein c)	0	KOG0054	K05665
ASTEI00096-RA	0,0,0	-0.545	0.056	-0.092	0.799	0.050	0.890	AGAP001777-PA	*An. gambiae*	ATP-binding cassette, sub-family C (CFTR/MRP), member 4	1E-71	NA	NA
ASTEI04513-RA	0,0,0	0.098	0.884	-0.464	0.465	-1.118	0.060	AGAP000506-PA	*An. gambiae*	ATP-binding cassette, sub-family G (WHITE), member 2b	0	KOG0061	NA
ASTEI07694-RA	0,0,0	-0.377	0.035	-0.439	0.013	-0.591	0.001	AGAP007917-PA	*An. gambiae*	ATP-binding cassette, subfamily C (CFTR/MRP), member 10	0	KOG0054	K05674
ASTEI09515-RA	0,0,0	-0.333	0.093	-0.277	0.187	-0.001	0.997	AGAP003221-PA	*An. gambiae*	ATP-binding cassette, sub-family C (CFTR/MRP), member 4	0	KOG0054	K05673
ASTEI09638-RA	0,0,0	0.042	0.785	-0.677	0.000	-0.380	0.005	AGAP009471-PA	*An. gambiae*	ATP-binding cassette sub-family G member 1	0	KOG0065	NA
ASTEI06493-RA	0,0,0	0.371	0.379	0.226	0.629	0.642	0.125	ASTEI06493-RA	*Ae. aegypti*	sulfonylurea receptor/ ABC transporter	0	KOG0054	K05033
ASTEI10920-RA	0,0,0	0.302	0.078	0.313	0.071	0.428	0.016	AGAP006364-PB	*An. gambiae*	ATP-binding cassette, subfamily B (MDR/TAP), member 7	0	KOG0057	K05662
ASTEI06540-RA	0,0,0	-0.066	0.798	-0.124	0.633	-0.158	0.573	AGAP009850-PA	*An. gambiae*	ATP-binding cassette sub-familyG member4	0	KOG0061	NA
ASTEI07390-RA	0,0,0	0.951	1.5e-08	0.118	0.590	-0.700	0.001	AGAP009463-PA	*An. gambiae*	ATP-binding cassette sub-family G member 1	0	KOG0061	NA
ASTEI01604-RA	0,0,1	0.823	0.029	0.172	0.729	1.071	0.004	AGAP007655-PA	*An. gambiae*	Brown protein	0	KOG0061	NA
ASTEI00154-RA	1,0,0	1.057	0.000	0.588	0.000	-0.360	0.004	AGAP002278-PA	*An. gambiae*	ATP-binding cassette, subfamily B (MDR/TAP), member 6	0	KOG0056	K05661
ASTEI04427-RA	1,1,1	1.880	0.000	1.844	0.000	1.566	0.000	ADAC008037-PA	*An. darlingi*	ABC membrane transporter	0	KOG0061	NA
ASTEI05756-RA	1,1,1	1.680	0.000	3.538	0.000	3.667	0.000	AGAP001333-PA	*An. gambiae*	Scarlet protein	0	KOG0061	NA

Phase I

CYPs

ASTEI00300-RA	-1,-1,-1	-1.837	0.000	-1.125	0.000	-1.329	0.000	AGAP003065-PA	*An. gambiae*	cytochrome P450_CYP11179	0	KOG1667	NA
ASTEI02928-RA	-1,-1,-1	-1.777	0.000	-2.755	0.000	-3.465	0.000	AGAP028019-PA	*An. gambiae*	cytochrome P450_CYP4H18	0	KOG0157	NA
ASTEI02931-RA	-1,-1,-1	-1.769	0.000	-1.471	0.001	-1.999	0.000	AGAP008356-PA	*An. gambiae*	cytochrome P450_CYP4H16	0	KOG0157	NA
ASTEI05843-RA	-1,-1,-1	-1.866	0.001	-2.647	0.000	-4.289	0.000	AGAP011029-PA	*An. gambiae*	cytochrome P450_CYP6AF2	0	KOG0158	K14999
ASTEI02248-RA	-1,-1,0	-1.660	0.000	-1.826	0.000	-0.726	0.002	AGAP005656-PA	*An. gambiae*	cytochrome P450_CYP305A1	0	KOG0156	NA
ASTEI00094-RA	-1,0,-1	-1.085	0.002	-0.882	0.014	-1.134	0.003	AGAP002894-PA	*An. gambiae*	cytochrome P450_CYP6Z4	0	KOG0158	K14999
ASTEI03053-RA	-1,0,-1	-1.789	0.000	-0.229	0.357	-1.789	0.000	AGAP008218-PA	*An. gambiae*	cytochrome P450_CYP6Z3	0	KOG0158	K14999
ASTEI07451-RA	-1,0,0	-1.485	0.004	0.466	0.427	-0.277	0.697	CPIJ012484-PA	*C. quinquefasciatus*	cytochrome P450_CYP6CP1	1E-27	NA	NA
ASTEI06914-RA	-1,0,1	-1.770	0.002	0.383	0.561	1.704	0.006	AAEL004870-PA	*Ae. aegypti*	cytochrome P450_CYP18A1	0	KOG0156	K14985
ASTEI01783-RA	0,-1,-1	-0.332	0.230	-1.164	0.007	-1.498	0.000	AGAP008022-PA	*An. gambiae*	cytochrome P450_CYP12F1	0	KOG0159	K15004
ASTEI11185-RA	0,-1,-1	-0.175	0.427	-1.672	0.000	-2.266	0.000	AGAP010966-PA	*An. gambiae*	cytochrome P450_CYP6AJ1	0	KOG0158	NA
ASTEI11577-RA	0,-1,-1	0.150	0.412	-1.062	0.000	-1.665	0.000	AGAP009363-PA	*An. gambiae*	cytochrome P450_CYP9M1	0	KOG0158	K15003
ASTEI09580-RA	0,-1,0	-1.178	0.064	-1.328	0.042	0.052	0.957	AGAP008682-PA	*An. gambiae*	cytochrome P450_CYP307B1	0	KOG0156	K14939
ASTEI00850-RA	0,0,-1	0.295	0.024	-0.771	0.000	-1.222	0.000	AGAP003343-PA	*An. gambiae*	cytochrome P450_CYP6AG1	0	KOG0158	K14999
ASTEI01072-RA	0,0,-1	0.814	0.000	-0.035	0.860	-1.362	0.000	AGAP000818-PA	*An. gambiae*	cytochrome P450_CYP9K1	0	KOG0158	K15003
ASTEI01187-RA	0,0,-1	0.946	0.257	0.000	1.000	-9.021	0.000	AGAP000877-PA	*An. gambiae*	cytochrome P450_CYP4G17	0	KOG0157	K15001
ASTEI02412-RA	0,0,-1	0.955	0.000	-0.517	0.008	-1.476	0.000	AGAP012296-PA	*An. gambiae*	cytochrome P450_CYP9J5	0	KOG0158	K15003
ASTEI02927-RA	0,0,-1	-0.106	0.481	-0.798	0.000	-1.228	0.000	AGAP008358-PA	*An. gambiae*	cytochrome P450_CYP4	0	KOG0157	NA
ASTEI03059-RA	0,0,-1	-0.499	0.018	-0.840	0.000	-1.467	0.000	AGAP008207-PA	*An. gambiae*	cytochrome P450_CYP6Y2	0	KOG0158	K14999
ASTEI08755-RA	0,0,-1	0.436	0.001	-0.210	0.141	-1.073	0.000	AGAP001864-PA	*An. gambiae*	cytochrome P450_CYP4G16	0	KOG0157	NA
ASTEI08938-RA	0,0,-1	0.948	0.000	-0.236	0.021	-1.083	0.000	AGAP013241-PA	*An. gambiae*	cytochrome P450_CYP4D16	0	KOG0157	NA
ASTEI00453-RA	0,0,0	0.591	0.000	-0.069	0.504	-0.516	0.000	AGAP002866-PA	*An. gambiae*	cytochrome P450_CYP6P4	0	KOG0158	NA
ASTEI02415-RA	0,0,0	0.321	0.010	-0.004	0.980	-0.520	0.000	AGAP012292-PA	*An. gambiae*	cytochrome P450_CYP9J4	0	KOG0158	NA
ASTEI03056-RA	0,0,0	0.244	0.027	-0.135	0.276	-0.840	0.000	AGAP008213-PA	*An. gambiae*	cytochrome P450_CYP6M3	0	KOG0158	NA
ASTEI03060-RA	0,0,0	0.110	0.605	0.781	0.000	0.069	0.764	AGAP008206-PA	*An. gambiae*	cytochrome P450_CYP6N2	0	KOG0158	K14999
ASTEI06230-RA	0,0,0	0.689	0.006	-0.481	0.078	-0.473	0.077	AGAP013490-PA	*An. gambiae*	cytochrome P450_CYP4H24	0	KOG0157	K15001
ASTEI06609-RA	0,0,0	0.802	0.003	0.860	0.002	0.747	0.007	AGAP009240-PA	*An. gambiae*	cytochrome P450_CYP4C35	0	KOG0157	NA
ASTEI07775-RA	0,0,0	0.362	0.016	0.591	0.000	0.353	0.035	AGAP001076-PA	*An. gambiae*	cytochrome P450_CYP4G16	0	KOG0157	K15001
ASTEI09341-RA	0,0,0	-0.001	0.997	-0.647	0.000	-0.386	0.037	AGAP003522-PA	*An. gambiae*	cytochrome P450_CYP329A1	0	KOG0158	NA
ASTEI10310-RA	0,0,0	0.597	0.000	-0.197	0.282	-0.710	0.000	AGAP009363-PA	*An. gambiae*	cytochrome P450_CYP9M1	1E-154	KOG0158	NA
ASTEI10596-RA	0,0,0	-1.342	0.058	0.287	0.747	0.706	0.390	AGAP000194-PA	*An. gambiae*	cytochrome P450_CYP4C25	0	KOG0157	NA
ASTEI10901-RA	0,0,0	0.981	0.000	0.034	0.922	-0.741	0.005	AGAP006047-PA	*An. gambiae*	cytochrome P450_CYP4J9	0	KOG0157	NA
ASTEI00597-RA	0,0,1	0.848	0.017	0.734	0.048	1.411	0.000	AGAP002205-PA	*An. gambiae*	cytochrome P450_CYP325C2	0	KOG0157	NA
ASTEI03057-RA	0,1,0	0.350	0.058	1.229	0.000	0.463	0.013	AAEL009124-PA	*Ae. aegypti*	cytochrome P450_CYP6N12	0	KOG0158	K14999

ASTEI00598-RA	0,1,1	0.491	0.388	1.392	0.006	1.518	0.002	AGAP002211-PA	*An. gambiae*	cytochrome P450_CYP325A1	0	KOG0157	NA
ASTEI08758-RA	1,0,-1	1.023	0.000	-0.487	0.001	-1.645	0.000	AGAP001861-PA	*An. gambiae*	cytochrome P450_CYP4H14	0	KOG0157	NA
ASTEI01400-RA	1,0,0	1.051	0.000	0.186	0.092	-0.184	0.132	AGAP007480-PA	*An. gambiae*	cytochrome P450_CYP6AH1	0	KOG0158	K20474
ASTEI08183-RA	1,0,0	1.301	0.000	0.740	0.001	0.351	0.207	AGAP003608-PA	*An. gambiae*	cytochrome P450_CYP4AA1	0	KOG0157	K15001
ASTEI08939-RA	1,0,0	1.095	0.000	0.708	0.001	0.144	0.595	AGAP002417-PA	*An. gambiae*	cytochrome P450_CYP4AR1	0	KOG0157	NA
ASTEI09029-RA	1,0,0	1.174	0.000	0.505	0.010	0.172	0.480	AGAP010961-PA	*An. gambiae*	cytochrome P450_CYP6AK1	0	KOG0158	K14999
ASTEI08940-RA	1,1,0	2.186	0.000	1.711	0.000	0.552	0.006	AGAP002416-PA	*An. gambiae*	cytochrome P450_CYP4K2	0	KOG0157	NA
ASTEI09473-RA	1,1,0	1.463	0.000	1.054	0.000	0.743	0.003	AGAP010414-PA	*An. gambiae*	cytochrome P450_CYP4C28	0	KOG0157	NA
ASTEI00299-RA	1,1,1	2.599	0.000	2.688	0.000	2.754	0.000	AGAP003066-PA	*An. gambiae*	cytochrome P450_CYP304B1	0	KOG0156	NA
ASTEI06605-RA	1,1,1	1.213	0.002	2.016	0.000	2.459	0.000	AGAP009246-PA	*An. gambiae*	cytochrome P450_CYP4C27	0	KOG0157	NA
AKRs													
ASTEI05819-RA	0,0,0	0.252	0.229	0.505	0.010	0.059	0.828	AAEL004086-PB	*Ae. aegypti*	aldo-ketoreductase	3E-173	KOG1577	K00011
EHs													
ASTEI09322-RA	0,0,0	0.364	0.036	0.236	0.216	-0.346	0.061	AGAP003542-PA	*An. gambiae*	Epoxide hydrolase 2, cytoplasmic, isoform CRA_a		KOG4178	NA
CCEs													
ASTEI06404-RA	-3	-1.294	0.000	-2.007	0.000	-1.818	0.000	AGAP010917-PA	*An. gambiae*	carboxylesterase	0	KOG1516	NA
ASTEI08528-RA	-1,0,-1	-1.770	0.001	-0.519	0.392	-1.314	0.022	AGAP005833-PA	*An. gambiae*	Carboxylesterase juvenile hormone esterase	0	KOG1516	NA
ASTEI00521-RA	0,-1,-1	-0.801	0.000	-1.392	0.000	-1.631	0.000	AGAP013509-PA	*An. gambiae*	Carboxylesterase clade H, member1	0	KOG1516	NA
ASTEI10045-RA	0,-1,-1	0.179	0.380	-1.477	0.000	-2.117	0.000	AGAP006726-PA	*An. gambiae*	carboxylesterase	0	KOG4389	NA
ASTEI04268-RA	0,-1,1	-0.041	0.934	-1.473	0.000	1.352	0.001	AGAP011365-PA	*An. gambiae*	carboxylesterase	0	KOG1516	NA
ASTEI10026-RA	0,0,-1	0.300	0.751	-0.832	0.338	-6.529	0.000	AGAP006700-PA	*An. gambiae*	Carboxylesterase alpha esterase	0	KOG4389	NA
ASTEI02522-RA	0,0,0	0.548	0.129	0.872	0.011	0.726	0.059	AGAP005758-PA	*An. gambiae*	carboxylesterase	0	KOG4389	NA
ASTEI06403-RA	0,0,0	-0.005	0.976	-0.468	0.000	-0.748	0.000	AGAP010911-PA	*An. gambiae*	carboxylesterase	0	KOG1516	NA
ASTEI08970-RA	0,0,0	-1.436	0.061	0.283	0.773	0.344	0.720	AGAP002391-PA	*An. gambiae*	carboxylesterase	0	KOG1516	NA
ASTEI09120-RA	0,0,0	-0.756	0.000	-0.785	0.000	-0.804	0.000	AGAP006228-PA	*An. gambiae*	carboxylesterase	0	KOG1516	NA
ASTEI10044-RA	0,0,0	0.238	0.049	-0.135	0.313	-0.394	0.002	AGAP006723-PA	*An. gambiae*	carboxylesterase	0	KOG4389	NA
ASTEI10069-RA	0,0,0	-0.602	0.000	0.186	0.171	0.813	0.000	AGAP005371-PA	*An. gambiae*	carboxylesterase beta esterase	0	KOG1516	NA
ASTEI10744-RA	0,0,0	0.708	0.005	0.581	0.026	0.168	0.615	AGAP001101-PA	*An. gambiae*	carboxylesterase	0	KOG4389	NA
ASTEI11262-RA	0,0,0	0.707	0.000	0.556	0.003	0.045	0.866	AGAP005757-PA	*An. gambiae*	Carboxylesterase alpha esterase	0	KOG1516	NA
ASTEI11467-RA	0,0,0	0.668	0.077	0.696	0.062	0.895	0.024	AGAP005758-PA	*An. gambiae*	carboxylesterase	3,00E-109	KOG1516	NA
ASTEI00392-RA	1,0,0	1.261	0.001	0.665	0.117	0.487	0.260	AGAP002090-PA	*An. gambiae*	carboxylesterase	0	KOG4389	NA
ASTEI02618-RA	1,0,0	1.257	0.022	0.146	0.847	0.887	0.144	AGAP006956-PA	*An. gambiae*	carboxylesterase	0	KOG4389	NA
ASTEI03121-RA	1,1,0	1.909	0.000	1.051	0.000	0.088	0.616	AGAP001723-PA	*An. gambiae*	Carboxylesterase alpha esterase	0	KOG1516	NA

ASTEI08772-RA	1,1,0	1.631	0.000	1.421	0.000	0.972	0.000	AGAP011507-PA	*An. gambiae*	carboxylesterase	0	KOG1516	NA
ASTEI06752-RA	1,1,1	1.522	0.003	1.837	0.001	1.315	0.012	AGAP010390-PA	*An. gambiae*	carboxylesterase	0	KOG4389	NA
**Phase II**													
GSTs													
ASTEI10390-RA	-1,-1,-1	-1.044	0.000	-1.705	0.000	-1.317	0.000	AGAP005749-PA	*An. gambiae*	glutathione S-transferase omega class	5,00E-169	KOG0406	K00310
ASTEI11637-RA	-1,-1,-1	-1.188	0.008	-1.929	0.000	-2.933	0.000	AGAP009193-PA	*An. gambiae*	glutathione S-transferase epsilon class	4,00E-73	KOG0867	NA
ASTEI00999-RA	0,-1,-1	-0.683	0.000	-1.831	0.000	-1.475	0.000	AGAP000761-PA	*An. gambiae*	glutathione S-transferase theta class	1,00E-150	KOG0867	K00799
ASTEI03480-RA	0,-1,-1	-0.714	0.006	-1.260	0.000	-1.271	0.000	AGAP000888-PB	*An. gambiae*	glutathione S-transferase theta class	2,00E-136	KOG0867	K00799
ASTEI05223-RA	0,-1,-1	-0.718	0.001	-1.728	0.000	-1.476	0.000	AGAP009195-PA	*An. gambiae*	glutathione S-transferase epsilon class	4,00E-121	KOG0867	K00799
ASTEI05224-RA	0,-1,-1	-0.021	0.916	-1.716	0.000	-1.624	0.000	AGAP009191-PA	*An. gambiae*	glutathione S-transferase epsilon class	4,00E-149	KOG0867	K00799
ASTEI05225-RA	0,-1,-1	-0.739	0.001	-1.037	0.000	-1.116	0.000	AGAP009196-PA	*An. gambiae*	glutathione S-transferase epsilon class	1,00E-150	KOG0867	K00799
ASTEI05226-RA	0,-1,-1	-0.847	0.000	-2.088	0.000	-2.031	0.000	AGAP009197-PA	*An. gambiae*	glutathione S-transferase epsilon class	6,00E-150	KOG0867	K00799
ASTEI07626-RA	0,-1,-1	-0.694	0.002	-1.203	0.000	-1.674	0.000	AGAP004172-PA	*An. gambiae*	glutathione S-transferase delta class	2,00E-147	KOG0867	K00799
ASTEI08702-RA	0,-1,-1	-0.353	0.002	-1.251	0.000	-1.509	0.000	AGAP004379-PA	*An. gambiae*	glutathione S-transferase delta class	1,00E-150	KOG0867	K00799
ASTEI08916-RA	0,-1,-1	-0.594	0.000	-1.276	0.000	-1.366	0.000	AGAP003257-PA	*An. gambiae*	glutathione S-transferase	8,00E-149	KOG0867	K00799
ASTEI10592-RA	0,-1,-1	-0.652	0.002	-1.144	0.000	-1.165	0.000	AGAP000947-PA	*An. gambiae*	glutathione S-transferase	6,00E-146	KOG0867	K00799
ASTEI07618-RA	0,0,0	0.159	0.590	-0.085	0.801	-0.598	0.036	CPIJ002661-PA	*C. quinquefasciatus*	glutathione S-transferase 1-6	0	KOG0867	NA
ASTEI07627-RA	0,0,0	-0.888	0.000	-0.810	0.001	-0.035	0.924	AGAP004173-PA	*An. gambiae*	glutathione S-transferase delta class	2,00E-122	KOG0867	K00799
ASTEI08703-RA	0,0,0	0.123	0.355	-0.389	0.001	-0.462	0.000	AGAP004382-PA	*An. gambiae*	glutathione S-transferase delta class	1,00E-128	KOG0867	NA
ASTEI09484-RA	0,0,0	-0.647	0.000	-0.346	0.029	-0.656	0.000	AGAP010404-PA	*An. gambiae*	glutathione S-transferase	2,00E-146	KOG1695	K04097
ASTEI11664-RA	0,0,0	-0.015	0.957	0.081	0.771	0.247	0.321	AGAP009192-PA	*An. gambiae*	glutathione S-transferase epsilon class	4E-142	KOG0867	K00799
ASTEI00084-RA	0,0,0	0.088	0.841	-0.460	0.266	-0.047	0.919	AGAP002898-PA	*An. gambiae*	glutathione S-transferase zeta class	2E-43	KOG0868	K01800
ASTEI00091-RA	0,0,0	0.468	0.188	-0.183	0.677	0.129	0.777	AGAP002898-PB	*An. gambiae*	glutathione S-transferase zeta class	5E-110	KOG0868	K01800
**UGTs**													
ASTEI02575-RA	-1,-1,-1	-1.253	0.000	-2.120	0.000	-1.834	0.000	AGAP007029-PA	*An. gambiae*	glucosyl/glucuronosyl transferases	0	KOG1192	K00699
ASTEI00210-RA	0,-1,-1	-0.745	0.000	-1.574	0.000	-1.548	0.000	AGAP002327-PA	*An. gambiae*	glucosyl/glucuronosyl transferases	0	KOG1192	K00699
ASTEI02576-RA	0,-1,-1	-0.142	0.540	-1.089	0.000	-1.246	0.000	AGAP007028-PA	*An. gambiae*	glucosyl/glucuronosyl transferases	0	KOG1192	K00699
ASTEI07685-RA	0,-1,-1	-0.565	0.078	-1.174	0.000	-1.694	0.000	AAEL000687-PA	*Ae. aegypti*	glucosyl/glucuronosyl transferases	0	KOG1192	K00699
ASTEI11135-RA	0,-1,-1	-0.175	0.715	-1.304	0.002	-2.221	0.000	AGAP011564-PA	*An. gambiae*	Glucosyl/glucuronosyl transferases	0	KOG1192	K00699
ASTEI10392-RA	0,-1,0	-0.194	0.257	-1.015	0.000	-0.488	0.004	AGAP005753-PA	*An. gambiae*	glucosyl/glucuronosyl transferases	0	KOG1192	K00699
ASTEI03891-RA	0,0,-1	-0.143	0.790	-0.989	0.049	-1.898	0.000	AGAP011564-PA	*An. gambiae*	Glucosyl/glucuronosyl transferases	0	KOG1192	K00699
ASTEI08260-RA	0,0,-1	-0.124	0.437	-0.921	0.000	-1.514	0.000	AGAP005163-PA	*An. gambiae*	Glucosyl/glucuronosyl transferases	0	KOG1192	K00699

ASTEI08615-RA	0,0,-1	-0.219	0.169	-0.595	0.000	-1.220	0.000	AGAP006775-PA	*An. gambiae*	Glucosyl/glucuronosyl transferases	0	KOG1192	K00699
ASTEI01492-RA	0,0,0	0.198	0.315	0.254	0.197	-0.687	0.000	AGAP007589-PA	*An. gambiae*	Glucosyl/glucuronosyl transferases	0	KOG1192	K00699
ASTEI07688-RA	0,0,0	-0.615	0.000	-0.510	0.003	-0.578	0.002	AGAP007920-PA	*An. gambiae*	Glucuronosyl transferase	0	KOG1192	K00699
ASTEI10974-RA	0,0,0	-0.625	0.034	-0.873	0.003	-0.532	0.101	AGAP002801-PA	*An. gambiae*	Galactosyl galactosyl xylosyl protein 3-beta-glucuronosyltransferase 3	0	KOG1476	K10158
ASTEI00013-RA	1,1,1	1.598	0.000	1.113	0.000	1.614	0.000	AGAP002783-PA	*An. gambiae*	UDP glucuronosyl transferase 5 family, polypeptide A2	0	KOG1192	K00699

Antioxidant enzymes

ASTEI07113-RA	0,-1,-1	-0.641	0.000	-1.248	0.000	-1.311	0.000	AGAP010517-PA	*An. gambiae*	manganese-iron (Mn-Fe) superoxide dismutase	4E-162	KOG0867	K04564
ASTEI05480-RA	0,0,0	-0.656	0.103	-0.743	0.068	0.438	0.376	AAEL006271-PC	*Ae. aegypti*	copper-zinc (Cu-Zn) superoxide dismutase	0.000008	NA	NA
ASTEI06006-RA	0,0,0	0.135	0.736	0.426	0.230	0.693	0.060	AGAP005234-PA	*An. gambiae*	copper-zinc (Cu-Zn) superoxide dismutase	1E-137	KOG0441	K04565
ASTEI06711-RA	0,0,0	0.157	0.114	-0.257	0.007	-0.486	0.000	AGAP010347-PA	*An. gambiae*	copper-zinc (Cu-Zn) superoxide dismutase	2E-96	KOG0441	K04565
ASTEI10644-RA	0,0,0	0.399	0.000	0.133	0.242	-0.449	0.000	AGAP004904-PA	*An. gambiae*	catalase	0	KOG0047	K03781
ASTEI06040-RA	1,1,1	1.838	0.000	1.927	0.000	1.686	0.000	CPIJ014001-PA	*C. quinquefasciatus*	Superoxide dismutase, Mn	0.000003	NA	NA

HSPs

ASTEI02621-RA	-1,-1,-1	-3.784	0.000	-1.522	0.000	-1.037	0.000	AAEL014845-PA	*Ae. aegypti*	heat shock protein	3E-99	KOG0019	K04079
ASTEI02622-RA	-1,-1,-1	-4.074	0.000	-1.549	0.000	-1.064	0.000	AGAP006958-PA	*An. gambiae*	Heat shock protein83	0	KOG0020	K04079
ASTEI06698-RA	-1,-1,-1	-2.319	0.000	-1.510	0.000	-1.706	0.000	AGAP010331-PA	*An. gambiae*	heat shock protein 110kDa	0	KOG0103	K09485
ASTEI07645-RA	-1,-1,-1	-1.903	0.000	-1.980	0.000	-1.109	0.000	AGAP004192-PA	*An. gambiae*	heat shock 70kDa protein 5	0	KOG0100	K09490
ASTEI11432-RA	-1,-1,-1	-4.029	0.000	-1.666	0.000	-1.018	0.000	AGAP006958-PA	*An. gambiae*	Heat shock protein83	0	KOG0020	K04079
ASTEI03620-RA	-1,-1,0	-2.068	0.000	-1.586	0.000	-0.815	0.000	AGAP001424-PA	*An. gambiae*	heat shock protein 90kDa beta	0	KOG0020	K09487
ASTEI04786-RA	-1,0,0	-2.969	0.000	0.818	0.016	0.336	0.419	AAEL017973-PA	*Ae. aegypti*	heat shock protein HSP70	0	KOG0101	K03283
ASTEI01441-RA	0,0,0	-0.164	0.042	-0.499	0.000	-0.780	0.000	AGAP013228-PA	*An. gambiae*	Heat shock protein 67B2	7E-74	KOG1530	NA
ASTEI05419-RA	0,0,0	0.317	0.019	0.264	0.057	0.275	0.079	AGAP002076-PA	*An. gambiae*	heat shock 70kDa protein 1/8	0	KOG0101	K03283
ASTEI10589-RA	0,0,0	-0.667	0.000	-0.555	0.000	-0.788	0.000	CPIJ009818-PA	*C. quinquefasciatus*	heat shock protein	7E-65	KOG3591	NA

CPs

ASTEI02027-RA	-1,0,0	-1.634	0.033	0.064	0.952	0.778	0.374	AGAP009758-PA	*An. gambiae*	Cuticular protein CPLCP11	1E-117	NA	NA
ASTEI02028-RA	-1,0,0	-1.869	0.017	0.393	0.689	0.847	0.332	AGAP009759-PA	*An. gambiae*	Cuticular protein CPLCP12	1E-135	NA	NA
ASTEI02077-RA	-1,0,0	-1.726	0.028	-0.066	0.952	0.620	0.502	AGAP005456-PA	*An. gambiae*	Cuticular protein 15 RR-1 family	3E-85	NA	NA
ASTEI02563-RA	-1,0,0	-1.865	0.017	0.324	0.748	0.367	0.706	AGAP007042-PA	*An. gambiae*	Cuticular protein RR-1 family (CPR62)	4E-100	NA	NA
ASTEI02890-RA	-1,0,0	-1.877	0.015	-1.020	0.223	0.338	0.722	AGAP006095-PA	*An. gambiae*	Cuticular protein RR-1 family (CPR106)	2E-97	NA	NA

ASTEI04631-RA	-1,0,0	-1.589	0.042	-0.131	0.905	0.332	0.724	AGAP006867-PA	*An. gambiae*	Cuticular protein RR-1 family (CPR141)	0	NA	NA
ASTEI04633-RA	-1,0,0	-2.420	0.001	0.075	0.944	0.000	1.000	AGAP006865-PA	*An. gambiae*	Cuticular protein RR-1 family (CPR64)	4E-78	NA	NA
ASTEI04634-RA	-1,0,0	-1.657	0.035	1.335	0.100	0.190	0.846	AGAP006847-PA	*An. gambiae*	Cuticular protein RR-1 family (CPR48)	6E-32	NA	NA
ASTEI04644-RA	-1,0,0	-2.642	0.000	0.029	0.978	0.444	0.642	AGAP006828-PA	*An. gambiae*	Cuticular protein RR-1 family (CPR60)	7E-35	NA	NA
ASTEI04792-RA	-1,0,0	-2.349	0.002	0.545	0.556	0.319	0.744	AGAP004576-PA	*An. gambiae*	Cuticular protein12 in TWDL family	3E-110	NA	NA
ASTEI04959-RA	-1,0,0	-2.339	0.002	0.102	0.927	0.430	0.649	AGAP028002-PA	*An. gambiae*	Cuticular protein (putative) CPLCP7	6E-172	NA	NA
ASTEI04960-RA	-1,0,0	-1.936	0.012	0.131	0.905	0.392	0.678	AGAP028042-PA	*An. gambiae*	Cuticular protien (putative) CPLCP6	6E-78	NA	NA
ASTEI04961-RA	-1,0,0	-2.890	0.000	-0.749	0.375	0.365	0.709	AGAP028018-PA	*An. gambiae*	Cuticular protein (putative) CPLCP24	2E-27	NA	NA
ASTEI05164-RA	-1,0,0	-1.777	0.009	-0.109	0.907	-0.586	0.480	AGAP010717-PA	*An. gambiae*	Cuticular protein RR-2 family (CPR143)	0	NA	NA
ASTEI05444-RA	-1,0,0	-1.811	0.020	0.155	0.887	0.595	0.511	AGAP013269-PA	*An. gambiae*	Cuticular protein10 in TWDL family	3E-159	NA	NA
ASTEI06161-RA	-1,0,0	-2.620	0.000	-0.174	0.873	0.000	1.000	AGAP000345-PA	*An. gambiae*	Cuticular protein RR-2 family (CPR126)	0.00002	NA	NA
ASTEI06228-RA	-1,0,0	-1.354	0.002	-0.407	0.428	0.947	0.060	AGAP000085-PA	*An. gambiae*	Cuticular protein RR-2 family (CPR129)	1E-148	NA	NA
ASTEI06313-RA	-1,0,0	-1.890	0.014	-0.188	0.860	0.321	0.735	AGAP006013-PA	*An. gambiae*	Cuticular protein RR-1 family (CPR33)	6E-97	NA	NA
ASTEI03708-RA	0,0,0	-1.388	0.085	-0.009	0.994	0.539	0.564	AGAP028191-PA	*An. gambiae*	Cuticular protein CPLCW family (CPLCW1)	2E-65	NA	NA
ASTEI03708-RA	0,0,0	-1.388	0.085	-0.009	0.994	0.539	0.564	AGAP028191-PA	*An. gambiae*	Cuticular protein CPLCW family (CPLCW1)	2E-65	NA	NA
ASTEI06558-RA	-1,0,0	-1.645	0.033	-0.012	0.992	1.142	0.169	AGAP009868-PA	*An. gambiae*	Cuticular protein RR-1 family (CPR73)	5E-71	NA	NA
ASTEI06559-RA	-1,0,0	-1.566	0.048	-0.030	0.978	0.279	0.771	AGAP009870-PA	*An. gambiae*	Cuticular protein 151	1E-76	NA	NA
ASTEI06684-RA	-1,0,0	-2.493	0.001	0.090	0.934	0.499	0.595	AGAP028124-PA	*An. gambiae*	Cuticular protein (putative) CPLCP2	2E-19	NA	NA
ASTEI06823-RA	-1,0,0	-2.559	0.001	-0.120	0.913	0.000	1.000	AGAP013465-PA	*An. gambiae*	Cuticular protein (putative) CPLCP1	0.0000000 6	NA	NA
ASTEI06998-RA	-1,0,0	-2.011	0.003	0.185	0.847	0.112	0.905	AGAP000344-PB	*An. gambiae*	Cuticular protein RR-1 family (CPR127)	8E-12	NA	NA
ASTEI07052-RA	-1,0,0	-1.762	0.023	-0.019	0.986	0.411	0.656	AGAP000352-PA	*An. gambiae*	Cuticular protein TWDL family (TWDL1)	9E-77	NA	NA
ASTEI08986-RA	-1,0,0	-1.644	0.038	-0.160	0.884	-0.016	0.989	AGAP010900-PA	*An. gambiae*	Cuticular protein1 from fifty-one aa family	1E-115	NA	NA
ASTEI09270-RA	-1,0,0	-1.655	0.022	-1.001	0.193	1.503	0.051	AGAP006321-PA	*An. gambiae*	Cuticular protein 71 RR-2 family	1E-97	NA	NA
ASTEI10168-RA	-1,0,0	-2.403	0.002	-0.345	0.730	0.395	0.676	AGAP006868-PB	*An. gambiae*	Cuticular protein RR-1 family (CPR140)	0.0000000 2	NA	NA
ASTEI10481-RA	-1,0,0	-2.302	0.002	-1.128	0.169	0.355	0.717	AGAP028042-PA	*An. gambiae*	Cuticular protien (putative) CPLCP6	2E-22	NA	NA
ASTEI10495-RA	-1,0,0	-1.935	0.012	-0.669	0.450	0.657	0.473	AGAP006261-PA	*An. gambiae*	Cuticular protein 135 RR-2 family	2E-47	NA	NA
ASTEI10584-RA	-1,0,0	-2.001	0.010	0.047	0.964	0.383	0.686	AGAP000538-PA	*An. gambiae*	Cuticular protein TWDL family (TWDL9)	7E-106	NA	NA

ASTEI11502-RA	-1,0,0	-1.688	0.032	0.170	0.875	0.738	0.410	AGAP006830-PA	*An. gambiae*	Cuticular protein RR-1 family (CPR58)	3E-17	NA	NA
ASTEI11618-RA	-1,0,0	-3.206	0.000	-1.543	0.052	0.537	0.566	AGAP006012-PA	*An. gambiae*	Cuticular protein RR-1 family (CPR32)	1E-33	NA	NA
ASTEI11785-RA	-1,0,0	-3.084	0.000	-1.484	0.064	0.457	0.629	AGAP006012-PA	*An. gambiae*	Cuticular protein RR-1 family (CPR32)	2E-57	NA	NA
ASTEI01032-RA	0,-1,-1	0.778	0.005	-2.585	0.000	-3.175	0.000	AGAP013421-PA	*An. gambiae*	Cuticular protein3 in TWDL family	9E-72	NA	NA
ASTEI03713-RA	0,-1,-1	-0.824	0.052	-1.136	0.006	-1.066	0.010	AGAP008469-PA	*An. gambiae*	Cuticular protein CPLCG family (CPLCG21)	0	NA	NA
ASTEI06564-RA	0,-1,0	0.177	0.713	-1.608	0.000	-0.442	0.384	AGAP009878-PA	*An. gambiae*	Cuticular protein RR-1 family (CPR80)	5E-136	NA	NA
ASTEI03709-RA	0,0,-1	0.989	0.017	-0.644	0.141	-1.562	0.000	AGAP008465-PA	*An. gambiae*	Cuticular protein CPLCG family (CPLCG22)	2E-41	NA	NA
ASTEI05298-RA	0,0,-1	-1.089	0.092	-0.147	0.862	-1.941	0.002	AGAP008444-PA	*An. gambiae*	Cuticular protein CPLCG family (CPLCG1)	4E-50	NA	NA
ASTEI05300-RA	0,0,-1	0.690	0.287	-0.250	0.748	-1.699	0.005	AGAP008446-PA	*An. gambiae*	Cuticular protein CPLCG family (CPLCG3)	5E-56	NA	NA
ASTEI06316-RA	0,0,-1	-0.619	0.230	-0.338	0.550	-1.298	0.012	AGAP006006-PA	*An. gambiae*	Cuticular protein RR-1 family (CPR104)	6E-67	NA	NA
ASTEI06317-RA	0,0,-1	-0.317	0.374	-0.989	0.002	-1.431	0.000	AGAP006005-PA	*An. gambiae*	Cuticular protein RR-1 family (CPR103)	2E-60	NA	NA
ASTEI11099-RA	0,0,-1	0.012	0.988	0.177	0.829	-1.295	0.050	AGAP008460-PA	*An. gambiae*	Cuticular protein CPLCG family (CPLCG16)	8E-50	NA	NA
ASTEI11131-RA	0,0,-1	0.619	0.177	-0.052	0.933	-2.217	0.000	AGAP006011-PA	*An. gambiae*	Cuticular protein RR-1 family (CPR31)	4E-44	NA	NA
ASTEI11350-RA	0,0,-1	0.647	0.268	-0.108	0.886	-1.886	0.000	AGAP008446-PA	*An. gambiae*	Cuticular protein CPLCG family (CPLCG3)	5E-56	NA	NA
ASTEI02026-RA	0,0,0	-1.477	0.056	-0.110	0.917	1.165	0.156	AGAP027993-PA	*An. gambiae*	Cuticular protein (putative) CPLCP10	0	NA	NA
ASTEI02074-RA	0,0,0	-1.528	0.056	-0.054	0.961	0.273	0.780	AGAP005454-PA	*An. gambiae*	Cuticular protein 13 RR-1 family	3E-90	NA	NA
ASTEI02353-RA	0,0,0	-0.714	0.387	0.060	0.954	1.361	0.087	AGAP028013-PA	*An. gambiae*	Cuticular protein (putative) CPLCP25	4E-89	NA	NA
ASTEI02565-RA	0,0,0	-1.485	0.056	-0.760	0.375	0.284	0.758	AGAP007040-PA	*An. gambiae*	Cuticular protein RR-1 family (CPR61)	2E-94	NA	NA
ASTEI02745-RA	0,0,0	0.800	0.021	0.142	0.750	0.458	0.274	AGAP006148-PA	*An. gambiae*	Cuticular protein3 in CPLCA family	4E-54	NA	NA
ASTEI03387-RA	0,0,0	-1.366	0.086	0.061	0.955	0.335	0.721	AGAP003390-PA	*An. gambiae*	Cuticular protein RR-2 family (CPR124)	1E-13	NA	NA
ASTEI03708-RA	0,0,0	-1.388	0.085	-0.009	0.994	0.539	0.564	AGAP028191-PA	*An. gambiae*	Cuticular protein CPLCW family (CPLCW1)	2E-65	NA	NA
ASTEI04635-RA	0,0,0	-1.350	0.090	0.263	0.796	0.232	0.809	AGAP006839-PA	*An. gambiae*	Cuticular protein RR-1 family (CPR67)	5E-153	NA	NA
ASTEI06318-RA	0,0,0	-1.341	0.092	-0.870	0.298	-0.112	0.912	AGAP006002-PA	*An. gambiae*	Cuticular protein RR-1 family (CPR137)	9E-59	NA	NA
ASTEI06561-RA	0,0,0	-0.246	0.585	-0.681	0.089	-0.992	0.020	AGAP009872-PA	*An. gambiae*	Cuticular protein RR-1 family (CPR133)	2E-57	NA	NA
ASTEI06565-RA	0,0,0	0.231	0.397	0.551	0.019	0.998	0.000	AGAP009879-PA	*An. gambiae*	Cuticular protein RR-1 family (CPR81)	8E-74	NA	NA
ASTEI06678-RA	0,0,0	-1.489	0.063	0.281	0.785	0.612	0.507	AGAP012462-PA	*An. gambiae*	Cuticular protein RR-2 family (CPR147)	1E-94	NA	NA
ASTEI06999-RA	0,0,0	-1.413	0.072	0.626	0.475	0.738	0.405	AGAP000345-PA	*An. gambiae*	Cuticular protein RR-2 family	2E-14	NA	NA

										(CPR126)			
ASTEI09421-RA	0,0,0	-0.430	0.628	1.343	0.082	1.031	0.219	AGAP006369-PA	*An. gambiae*	Cuticular protein 144	0	NA	NA
ASTEI02354-RA	0,0,1	-1.055	0.151	-0.447	0.596	1.958	0.007	AGAP028137-PA	*An. gambiae*	Cuticular protein (putative) CPLCP17	8E-94	NA	NA
ASTEI02355-RA	0,0,1	-0.751	0.358	-0.200	0.845	1.685	0.029	AGAP028178-PA	*An. gambiae*	Cuticular protein (putative) CPLCP13	5E-100	NA	NA
ASTEI03181-RA	0,0,1	0.563	0.160	0.978	0.008	2.100	0.000	AGAP002726-PA	*An. gambiae*	Cuticular protein 9 RR-1 family	2E-99	NA	NA
ASTEI05035-RA	0,0,1	-1.198	0.119	0.117	0.911	1.693	0.027	AGAP008817-PA	*An. gambiae*	Cuticular protein (putative) CPLCP3	0	NA	NA
ASTEI08802-RA	0,0,1	0.273	0.690	0.764	0.209	1.420	0.020	AGAP000344-PB	*An. gambiae*	Cuticular protein RR-1 family (CPR127)	2E-121	NA	NA
ASTEI02593-RA	1,0,-1	1.656	0.001	-0.364	0.568	-1.530	0.006	AGAP006931-PA	*An. gambiae*	Cuticular protein 111 RR-3 family	1E-113	NA	NA
ASTEI01256-RA	1,0,0	1.009	0.001	0.322	0.339	-0.228	0.560	AGAP000047-PA	*An. gambiae*	Cuticular protein RR-2 family (CPR130)	1E-150	NA	NA
ASTEI06319-RA	1,0,0	1.193	0.011	0.201	0.744	-0.253	0.674	AGAP005995-PA	*An. gambiae*	Cuticular protein RR-1 family (CPR138)	0	NA	NA
ASTEI08996-RA	1,0,0	1.075	0.000	0.854	0.001	0.592	0.038	AGAP010887-PA	*An. gambiae*	Cuticular protein RR-2 family (CPR113)	2E-149	NA	NA
ASTEI10113-RA	1,0,0	1.217	0.002	0.506	0.245	-0.124	0.825	AGAP006497-PA	*An. gambiae*	Cuticular protein 134	0	NA	NA
**Ahr**													
ASTEI09831-RA	0,0,-1	-0.390	0.363	-0.041	0.940	-1.760	0.000	AAEL011825-PC	*Ae. aegypti*	Aryl hydrocarbon receptor	0	KOG3560	NA
ASTEI02023-RA	0,0,0	-0.128	0.473	0.238	0.154	0.277	0.085	AGAP009748-PA	*An. gambiae*	aryl hydrocarbon receptor nuclear translocator	0	KOG3561	K09097
ASTEI04432-RA	0,1,0	0.794	0.000	1.065	0.000	0.811	0.000	AGAP000725-PA	*An. gambiae*	Aryl hydrocarbon receptor interacting protein	0	KOG0545	K17767
**NRs**													
ASTEI02952-RA	-1,0,0	-1.382	0.037	0.261	0.756	-0.115	0.895	AGAP008334-PA	*An. gambiae*	nuclear receptor subfamily 4 group A member 2	0	KOG4217	K08558
ASTEI08516-RA	0,-1,0	0.265	0.699	-2.382	0.000	-0.873	0.255	AGAP006571-PA	*An. gambiae*	nuclear receptor subfamily 1 group D member 3	7E-56	NA	NA
ASTEI02478-RA	0,0,0	-0.110	0.752	-0.259	0.431	0.025	0.955	AAEL007397-PC	*Ae. aegypti*	Ecdysone-induced protein 75B isoform A Nuclear receptor	0	KOG4216	K08701
ASTEI02479-RA	0,0,0	-0.752	0.228	-0.359	0.613	-0.181	0.827	AGAP012223-PA	*An. gambiae*	nuclear receptor subfamily 1 group D member 3	1E-52	KOG4846	NA
ASTEI07992-RA	0,0,0	-0.324	0.523	-0.282	0.589	-0.594	0.214	AGAP002544-PA	*An. gambiae*	nuclear receptor subfamily 2 group F member 3	6E-97	NA	NA
ASTEI07993-RA	0,0,0	-0.537	0.350	-0.125	0.858	-0.300	0.644	AGAP002544-PB	*An. gambiae*	nuclear receptor subfamily 2 group F member 3	0	KOG4215	NA
ASTEI07994-RA	0,0,0	-0.137	0.847	0.112	0.897	0.099	0.898	AAEL002765-PA	*Ae. aegypti*	Seven up nuclear receptor	4E-59	KOG4215	NA
ASTEI08329-RA	0,0,0	-0.356	0.585	0.594	0.355	0.579	0.407	AGAP010438-PA	*An. gambiae*	nuclear receptor subfamily 0 group A	3E-38	KOG4216	K08706

ASTEI08836-RA	0,0,0	0.611	0.355	0.466	0.518	0.342	0.649	AGAP000981-PA	*An. gambiae*	Nuclear receptor interaction protein	3E-53	KOG1310	K11795
ASTEI10441-RA	0,0,0	-1.190	0.116	-0.008	0.994	-0.252	0.796	AGAP004693-PA	*An. gambiae*	nuclear receptor subfamily 6 group A	4E-53	NA	NA
ASTEI10442-RA	0,0,0	-1.051	0.197	-0.148	0.891	-0.009	0.993	AGAP004693-PA	*An. gambiae*	nuclear receptor subfamily 6 group A	0	KOG4218	K09185
ASTEI06629-RA	0,0,0	0.211	0.239	-0.010	0.966	0.435	0.009	AGAP008382-PA	*An. gambiae*	nuclear receptor subfamily 2 group C	0	KOG4215	NA
ASTEI01071-RA	0,0,0	1.029	0.122	1.141	0.086	0.350	0.674	AGAP000819-PA	*An. gambiae*	nuclear receptor subfamily 2 group E member (Tailless)	0	KOG4215	K08545
ASTEI07347-RA	0,0,0	-0.343	0.105	-0.577	0.004	-0.435	0.054	AGAP009400-PA	*An. gambiae*	nuclear receptor subfamily 5 group A member 3	0	KOG4218	K08705
ASTEI10443-RA	0,0,0	-1.479	0.057	0.175	0.868	-0.099	0.923	AGAP004693-PA	*An. gambiae*	nuclear receptor subfamily 6 group A	8E-39	NA	NA
ASTEI03690-RA	0,0,0	0.542	0.001	-0.003	0.991	-0.183	0.325	AGAP004224-PA	*An. gambiae*	nuclear receptor subfamily 1 group I	0	KOG4215	K14035
ASTEI08331-RA	0,0,0	0.561	0.098	-0.526	0.152	0.146	0.763	AGAP010438-PA	*An. gambiae*	nuclear receptor subfamily 0 group A	0	KOG4216	NA
ASTEI04688-RA	0,0,0	0.364	0.005	0.243	0.078	0.050	0.775	AGAP009664-PA	*An. gambiae*	nuclearreceptorcoactivator2	0	NA	K11255
ASTEI01760-RA	0,0,0	0.773	0.000	0.818	0.000	0.385	0.022	AGAP007996-PB	*An. gambiae*	Nuclear receptor-binding protein	0	KOG1266	K08875
**MAPK**													
ASTEI01876-RA	0,0,0	0.161	0.799	-0.649	0.272	-0.497	0.436	AGAP008129-PA	*An. gambiae*	mitogen-activated protein kinase organizer 1	0	KOG0316	K13124
ASTEI03404-RA	0,0,0	-0.285	0.252	-0.211	0.435	0.071	0.821	AGAP003365-PA	*An. gambiae*	mitogen-activated protein kinase kinase 4	0	KOG1006	K04430
ASTEI10746-RA	0,0,0	0.096	0.726	-0.238	0.373	0.015	0.965	AGAP001103-PA	*An. gambiae*	mitogen-activated protein kinase kinase 1	0	KOG0581	K04368
ASTEI08749-RA	0,0,0	0.189	0.174	0.272	0.042	0.256	0.068	AGAP001867-PA	*An. gambiae*	mitogen-activated protein kinase kinase 7	0	KOG0983	K04431
ASTEI10965-RA	0,0,0	0.241	0.240	0.372	0.063	0.528	0.007	AGAP000310-PA	*An. gambiae*	mitogen-activated protein kinase kinase 3	0	KOG0984	K04432
ASTEI03516-RA	0,0,0	0.429	0.056	0.282	0.270	0.118	0.675	AGAP000997-PA	*An. gambiae*	mitogen-activated protein kinase kinase 1 interacting protein 1	2E-86	NA	K04370
ASTEI00503-RA	0,0,0	0.489	0.097	0.462	0.128	0.693	0.021	AGAP002953-PA	*An. gambiae*	Mitogen-activated protein kinase kinase kinase 7-interacting protein 1	0	NA	K04403
ASTEI02510-RA	0,0,0	0.494	0.000	0.386	0.006	0.063	0.717	AGAP010837-PA	*An. gambiae*	mitogen-activated protein kinase kinase kinase kinase 5	0	KOG0576	K08833
ASTEI04318-RA	0,0,0	0.550	0.000	0.231	0.059	0.127	0.370	CPIJ001610-PA	*C. quinquefasciatus*	mitogen-activated protein kinase kinasekinase 4	0	KOG4645	K04428

ASTEI05089-RA	0,0,0	0.336	0.088	0.330	0.093	0.616	0.001	AGAP011890-PA	*An. gambiae*	mitogen-activated protein kinase-activated protein kinase 2	8E-166	KOG0604	K04443
ASTEI10136-RA	0,0,0	0.618	0.000	0.731	0.000	0.387	0.002	AGAP006461-PA	*An. gambiae*	mitogen-activated protein kinase kinase kinase 13	0	KOG4721	K04422
ASTEI06997-RA	0,1,1	0.926	0.000	1.942	0.000	1.754	0.000	AGAP000747-PB	*An. gambiae*	mitogen-activated protein kinase kinase kinase 5	0	KOG4279	K04426
**Nrf2**													
ASTEI10187-RA	0,0,0	0.036	0.900	0.293	0.242	0.010	0.978	AGAP005300-PA	*An. gambiae*	Nuclear factor erythroid 2, invertebrate	0	NA	NA
ASTEI10188-RA	0,0,0	0.688	0.000	0.558	0.000	0.164	0.146	AGAP005300-PA	*An. gambiae*	Nuclear factor erythroid 2, invertebrate	0	KOG3863	K09041
ASTEI05762-RA	0,1,0	0.364	0.102	2.297	0.000	0.564	0.014	AGAP001324-PA	*An. gambiae*	Erythroiddifferentiation-relatedfactor1	0	NA	NA
**HSF**													
ASTEI05581-RA	0,0,0	0.101	0.528	0.306	0.030	0.376	0.011	AAEL010319-PA	*Ae. aegypti*	heat shock transcription factor (hsf)	2E-107	KOG0627	NA
**Others xenobiotics defense-related genes**													
ASTEI00237-RA	0,0,0	-0.268	0.488	-0.365	0.342	-0.308	0.457	AAEL008369-PA	*Ae. aegypti*	acyl phosphatase, putative	2E-56	KOG3360	K01512
ASTEI00250-RA	0,0,0	0.022	0.907	-0.540	0.000	-0.345	0.030	AGAP003124-PA	*An. gambiae*	dihydropyrimidinase	0	KOG2584	K01464
ASTEI00806-RA	0,0,0	0.253	0.252	0.718	0.000	0.689	0.001	AGAP003874-PC	*An. gambiae*	uridine kinase	0	KOG4203	K00876
ASTEI01275-RA	0,0,0	-0.605	0.459	0.207	0.836	0.485	0.564	AGAP007300-PA	*An. gambiae*	alkaline phosphatase	0	KOG4126	K01077
ASTEI01329-RA	0,0,0	0.355	0.560	0.288	0.683	0.441	0.514	AGAP007374-PA	*An. gambiae*	glucuronosyltransferase	0	KOG1192	K00699
ASTEI01662-RA	-1,-1,-1	-1.713	0.001	-2.430	0.000	-1.516	0.015	AGAP007793-PA	*An. gambiae*	Regucalcin protein	0	KOG4499	K00699
ASTEI01671-RA	0,-1,-1	-0.814	0.000	-1.716	0.000	-1.360	0.000	AGAP007784-PA	*An. gambiae*	enoyl-CoA hydratase / long-chain 3-hydroxyacyl-CoA dehydrogenase	0	KOG1683	K07515
ASTEI02231-RA	1,1,1	1.569	0.001	1.113	0.018	1.232	0.005	AGAP005637-PA	*An. gambiae*	aldehyde oxidase	0	KOG0430	K00106
ASTEI02232-RA	1,1,1	2.061	0.000	2.147	0.000	1.789	0.000	AGAP005638-PA	*An. gambiae*	aldehyde oxidase	0	KOG0430	K00106
ASTEI02833-RA	1,1,0	1.675	0.000	1.054	0.016	0.380	0.501	AGAP005865-PA	*An. gambiae*	fumarylacetoacetase	0	KOG2843	K01555
ASTEI02975-RA	0,0,-1	-0.670	0.046	0.087	0.851	-1.314	0.000	AGAP008311-PA	*An. gambiae*	acylphosphatase	7E-71	KOG3360	K01512
ASTEI03083-RA	0,-1,0	0.945	0.057	-1.457	0.003	0.168	0.810	AGAP001684-PA	*An. gambiae*	alkaline phosphatase	0	KOG4126	K01078
ASTEI03532-RA	0,-1,0	0.706	0.000	-1.215	0.000	-0.447	0.036	AGAP001021-PA	*An. gambiae*	dihydropyrimidine dehydrogenase (NADP+)	0	KOG1799	K00207

ASTEI04027-RA	0,-1,0	-0.756	0.074	-1.075	0.009	-0.521	0.270	AGAP009945-PA	*An. gambiae*	dihydrodiol dehydrogenase / D-xylose 1-dehydrogenase (NADP)	0	KOG2741	K00078
ASTEI04028-RA	0,0,-1	-0.030	0.878	-0.967	0.000	-1.508	0.000	AGAP009944-PA	*An. gambiae*	aldehyde dehydrogenase (NAD+)	0	KOG2450	K00128
ASTEI04208-RA	1,1,0	1.987	0.000	1.027	0.000	-0.488	0.004	AGAP011302-PA	*An. gambiae*	alkaline phosphatase	0	KOG4126	K01079
ASTEI04209-RA	1,1,0	1.261	0.000	1.501	0.000	0.246	0.196	AGAP011305-PB	*An. gambiae*	alkaline phosphatase	0	KOG4126	K01080
ASTEI04235-RA	0,-1,-1	-0.935	0.000	-1.837	0.000	-1.322	0.000	CPIJ019232-PA	*C. quinquefasciatus*	acetyl-CoA acetyltransferase, mitochondrial	0	KOG1390	K00626
ASTEI04371-RA	0,0,0	0.608	0.001	0.168	0.438	0.182	0.453	AGAP003578-PA	*An. gambiae*	aldehyde dehydrogenase (NAD+)	0	KOG2450	K00128
ASTEI04736-RA	1,0,0	1.619	0.000	0.763	0.087	0.342	0.540	AGAP009609-PA	*An. gambiae*	homogentisate 1,2-dioxygenase	0	KOG1417	K00451
ASTEI04740-RA	1,0,0	1.203	0.000	0.779	0.000	0.651	0.001	AGAP008501-PA	*An. gambiae*	glutaryl-CoA dehydrogenase	0	KOG0138	K00252
ASTEI04789-RA	0,0,-1	0.663	0.012	-0.631	0.028	-2.119	0.000	AGAP012634-PA	*An. gambiae*	Alkaline phosphatase	0	KOG4126	K01081
ASTEI05071-RA	0,0,0	0.560	0.000	-0.380	0.000	-0.815	0.000	AGAP011859-PA	*An. gambiae*	beta-glucuronidase	0	KOG2024	K01195
ASTEI05340-RA	0,0,0	-0.708	0.251	0.091	0.910	1.213	0.052	AGAP010596-PA	*An. gambiae*	alkaline phosphatase	0	KOG4126	K01082
ASTEI05639-RA	-1,0,0	-1.227	0.000	-0.616	0.001	-0.411	0.046	AGAP011133-PA	*An. gambiae*	Inosine-5-monophosphate dehydrogenase	0	KOG2550	K00088
ASTEI05718-RA	-1,0,0	-1.130	0.000	-0.240	0.342	-0.033	0.917	AGAP001501-PA	*An. gambiae*	Regucalcin protein	0	KOG4499	K01054
ASTEI05767-RA	-1,-1,-1	-1.183	0.000	-1.681	0.000	-1.067	0.000	AGAP001318-PA	*An. gambiae*	acetyl-CoA C-acetyltransferase	0	KOG1390	K00626
ASTEI06032-RA	0,0,0	0.284	0.088	0.487	0.003	0.301	0.095	AGAP005209-PA	*An. gambiae*	uridine kinase	0	KOG4203	K00876
ASTEI06079-RA	0,0,0	0.383	0.000	-0.269	0.021	-0.495	0.000	CPIJ006479-PA	*C. quinquefasciatus*	3-hydroxyacyl-coa dehyrogenase	0	KOG2304	K00022
ASTEI06183-RA	-1,0,0	-1.833	0.017	0.085	0.937	1.352	0.096	AGAP009137-PA	*An. gambiae*	Ecdysteroid UDP-glucosyltransferase	0	KOG1192	K00699
ASTEI06614-RA	-1,-1,-1	-1.399	0.000	-1.578	0.000	-1.297	0.000	CPIJ000349-PA	*C. quinquefasciatus*	UDP-glucuronosyltransferase 1-1	0	KOG1192	K00699
ASTEI06637-RA	0,-1,0	-0.849	0.000	-1.056	0.000	-0.678	0.003	AGAP008374-PA	*An. gambiae*	inosine triphosphate pyrophosphatase	1E-126	KOG3222	K01519
ASTEI06805-RA	1,1,1	1.409	0.000	1.866	0.000	1.190	0.000	CPIJ002095-PA	*C. quinquefasciatus*	alkaline phosphatase	9E-24	KOG4126	K01083
ASTEI02975-RA	0,0,-1	-0.670	0.046	0.087	0.851	-1.314	0.000	AGAP008311-PA	*An. gambiae*	acylphosphatase	7E-71	KOG0022	K01512
ASTEI03083-RA	0,-1,0	0.945	0.057	-1.457	0.003	0.168	0.810	AGAP001684-PA	*An. gambiae*	alkaline phosphatase	0	KOG2450	K01078
ASTEI03532-RA	0,-1,0	0.706	0.000	-1.215	0.000	-0.447	0.036	AGAP001021-PA	*An. gambiae*	dihydropyrimidine dehydrogenase (NADP+)	0	KOG1377	K00207
ASTEI04027-RA	0,-1,0	-0.756	0.074	-1.075	0.009	-0.521	0.270	AGAP009945-PA	*An. gambiae*	dihydrodiol dehydrogenase / D-xylose 1-dehydrogenase (NADP)	0	KOG0430	K00078
ASTEI04028-RA	0,0,-1	-0.030	0.878	-0.967	0.000	-1.508	0.000	AGAP009944-PA	*An. gambiae*	aldehyde dehydrogenase (NAD+)	0	KOG0430	K00128
ASTEI04208-RA	1,1,0	1.987	0.000	1.027	0.000	-0.488	0.004	AGAP011302-PA	*An. gambiae*	alkaline phosphatase	0	KOG0430	K01079
ASTEI04209-RA	1,1,0	1.261	0.000	1.501	0.000	0.246	0.196	AGAP011305-PB	*An. gambiae*	alkaline phosphatase	0	KOG0808	K01080

ASTEI04235-RA	0,-1,-1	-0.935	0.000	-1.837	0.000	-1.322	0.000	CPIJ019232-PA	*C. quinquefasciatus*	acetyl-CoA acetyltransferase, mitochondrial	0	KOG1622	K00626
ASTEI04371-RA	0,0,0	0.608	0.001	0.168	0.438	0.182	0.453	AGAP003578-PA	*An. gambiae*	aldehyde dehydrogenase (NAD+)	0	KOG0430	K00128
ASTEI04736-RA	1,0,0	1.619	0.000	0.763	0.087	0.342	0.540	AGAP009609-PA	*An. gambiae*	homogentisate 1,2-dioxygenase	0	KOG0430	K00451
ASTEI07133-RA	0,0,0	-0.413	0.001	-0.515	0.000	-0.243	0.108	AGAP010499-PA	NA	S-(hydroxymethyl)glutathione dehydrogenase /Alcohol dehydrogenase (Kegg orthology)	NA	KOG1680	K00121
ASTEI07414-RA	0,0,0	-0.512	0.000	-0.681	0.000	-0.720	0.000	AGAP003652-PB	*An. gambiae*	aldehyde dehydrogenase (NAD+)	0	KOG4126	K00128
ASTEI07629-RA	-1,0,0	-1.580	0.000	-0.943	0.000	-0.737	0.004	AGAP004175-PA	*An. gambiae*	uridine monophosphate synthetase	0	KOG3728	K13421
ASTEI07691-RA	0,0,0	0.475	0.003	0.724	0.000	0.272	0.148	AGAP007918-PA	*An. gambiae*	xanthine dehydrogenase/oxidase	0	KOG1192	K00106
ASTEI07692-RA	0,0,0	0.474	0.001	-0.012	0.953	-0.320	0.044	AGAP007918-PA	*An. gambiae*	xanthine dehydrogenase/oxidase	0	KOG0430	K00106
ASTEI07693-RA	0,0,0	0.475	0.043	0.563	0.022	0.193	0.517	AGAP007918-PA	*An. gambiae*	xanthine dehydrogenase/oxidase	6E-169	KOG2843	K00106
ASTEI07874-RA	1,1,0	1.773	0.000	1.960	0.000	0.980	0.000	AGAP010229-PA	*An. gambiae*	beta-ureidopropionase	0	KOG3360	K01431
ASTEI08316-RA	0,0,0	-0.375	0.002	-0.281	0.025	-0.071	0.651	AGAP010139-PD	*An. gambiae*	GMP synthase (glutamine-hydrolysing)	0	KOG4126	K01951
ASTEI09117-RA	1,1,0	1.548	0.000	1.020	0.002	0.730	0.052	AGAP006221-PA	*An. gambiae*	aldehyde oxidase	0	KOG1799	K00106
ASTEI09118-RA	0,0,0	-0.050	0.773	-0.358	0.011	0.289	0.073	AGAP006226-PA	*An. gambiae*	xanthine dehydrogenase/oxidase	0	KOG2741	K00106
ASTEI09239-RA	0,-1,-1	-0.681	0.000	-1.680	0.000	-1.306	0.000	AGAP011833-PA	*An. gambiae*	enoyl-CoA hydratase	0	KOG2450	K07511
ASTEI09453-RA	-1,-1,-1	-2.458	0.000	-3.334	0.000	-1.339	0.002	AGAP006400-PA	*An. gambiae*	Alkaline phosphatase 2	0	KOG4126	K01084
ASTEI10601-RA	0,-1,0	-0.659	0.000	-1.106	0.000	-0.484	0.017	AGAP000188-PA	*An. gambiae*	uridine phosphorylase	0	KOG4126	K00757
ASTEI11082-RA	-1,-1,-1	-1.204	0.000	-1.386	0.000	-1.600	0.000	CPIJ000351-PA	*C. quinquefasciatus*	UDP-glucuronosyltransferase 2B28	0	KOG1390	K00699

Table S5. Primers used in RT-qPCR validation. List of the primer pairs used to amplify the target genes selected to validate the transcriptomic results.

***An. stephensi* ID**	**Gene target**	**Forward primer (5'-3')**	**Reverse primer (5'-3')**	**PCR product size (base pairs)**	**Source**
ASTEI02233-RA	ABC-B2	TATCAAGTTCACGGATGTAGAGT	TATCCACCTTGCCACTGTC	185	[33]
ASTEI05756-RA	ABC-G4	ATGAGCCCATTCGTCCTG	AGCGTGGAGAAGAAGCAG	158	[33]
ASTEI08940-RA	CYP4K2	GCTGAGTTCCATCCTGTAC	TTTCCTCCTCCTCCTGCT	210	This study
ASTEI05843-RA	CYP6AF2	TTTTTACCGTTTGGGGAGGG	CCTGTGACTGTAGCGTAAC	169	This study
ASTEI06404-RA	CARB1	AGGGATCAAAGATGCGACA	CTCCATCAGATATTCGGC	219	This study
ASTEI06752-RA	CARB2	GAAACCTTGGTCTGCTCG	TGAAAAGATTGTGCCCGACATA	199	This study
ASTEI05226-RA	GST2	GAAAGATGACGCCCTCTAC	TCTGTCCCCAGTAAAACACC	135	This study
ASTEI01032-RA	CuPro3	GCCATCTCTAACTCGTTC	CCGAAACCACCATCGAAT	154	This study
	Rps7	AGCAGCAGCAGCACTTGATTTG	TAAACGGCTTTCTGCGTCACCC	90	[33]

Figure S1. Maximum likelyhood phylogenetic tree of ABC transporter family. Colors show the ABC transporter sub-families: ABCBA (green), ABCB (blue); ABCC (magenta); ABCD (red); ABCE (violet); ABCF (yellow); ABCG (brown). The code at the end of the branches represents the ID of each ABC transporter gene detected in *Anopheles stephensi*. The numbers on the branch node indicate the bootstrap values.

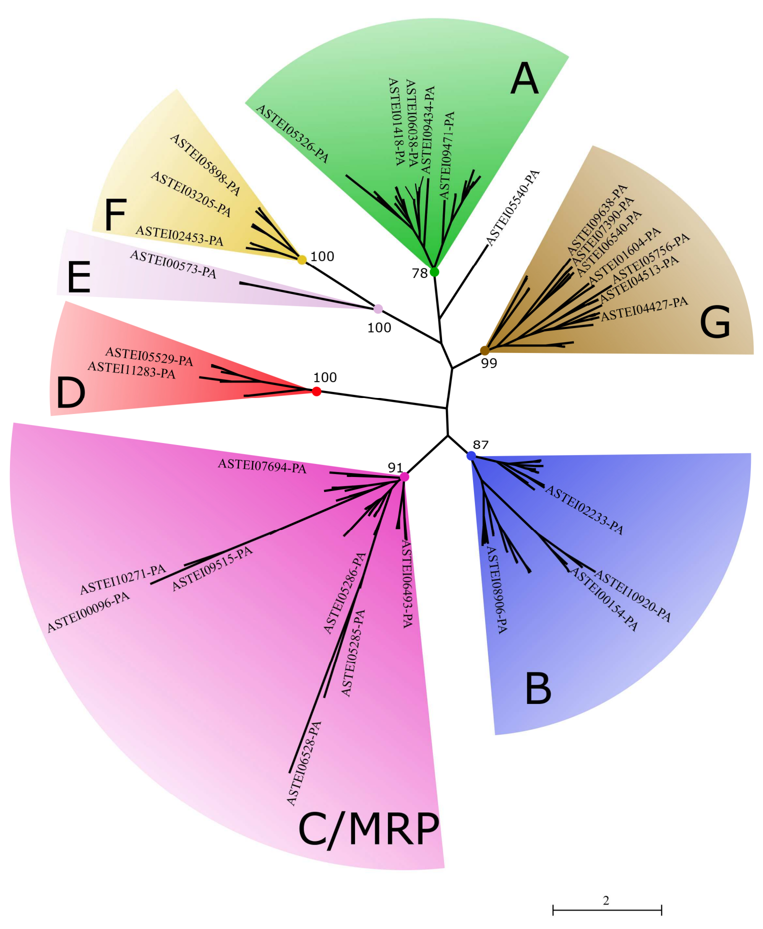

Figure S2. Venn diagrams. Proportions of *Anopheles stephensi* up- and down-regulated genes encoding for Phase I, Phase II, Phase 0/III enzymes and Cuticular Proteins (CPs) after six, 24 and 48 hours.

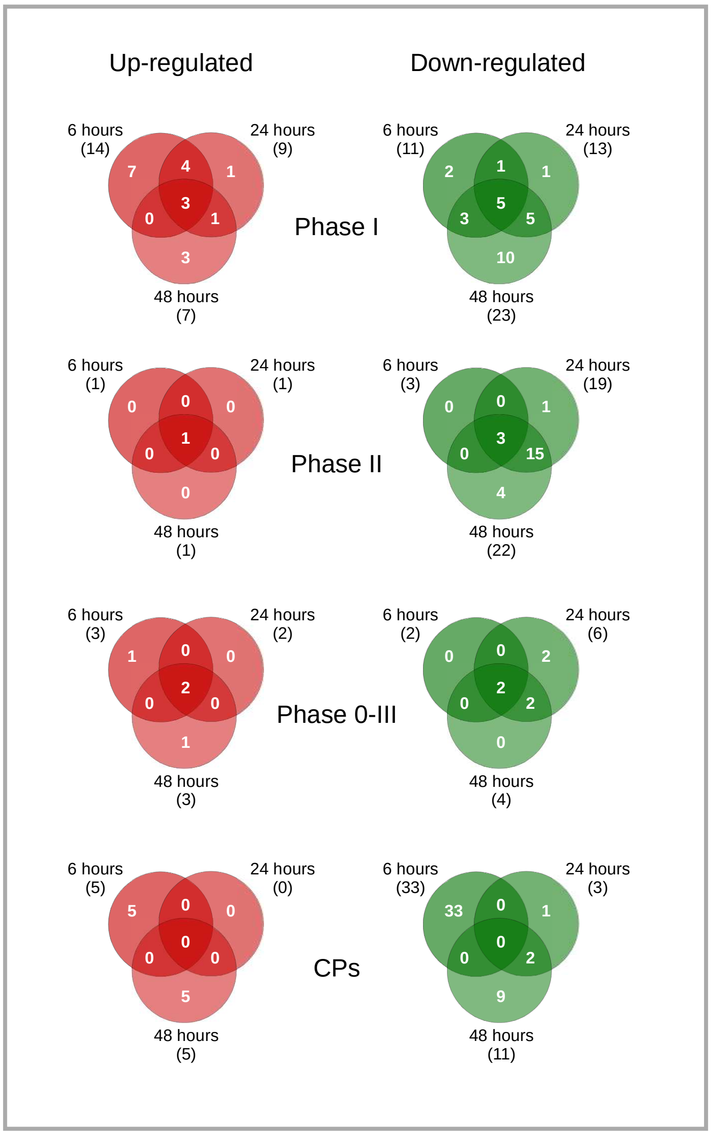

Figure S3. Cluster analysis of insecticide defense-related genes differentially expressed at six, 24 and 48h. Hierarchical clustering analysis based on log fold-change. Genes not differentially expressed at any time point were excluded from the analysis. For each gene, the ID is also indicated. Genes included in the analysis were: ABC transporters, CYPs, CCEs, AKRs, EH, GSTs, UGTs, antioxidant enzymes, HSPs, CPs and KEGG orthologs belonging to the “xenobiotics biodegradation and metabolism” pathways.

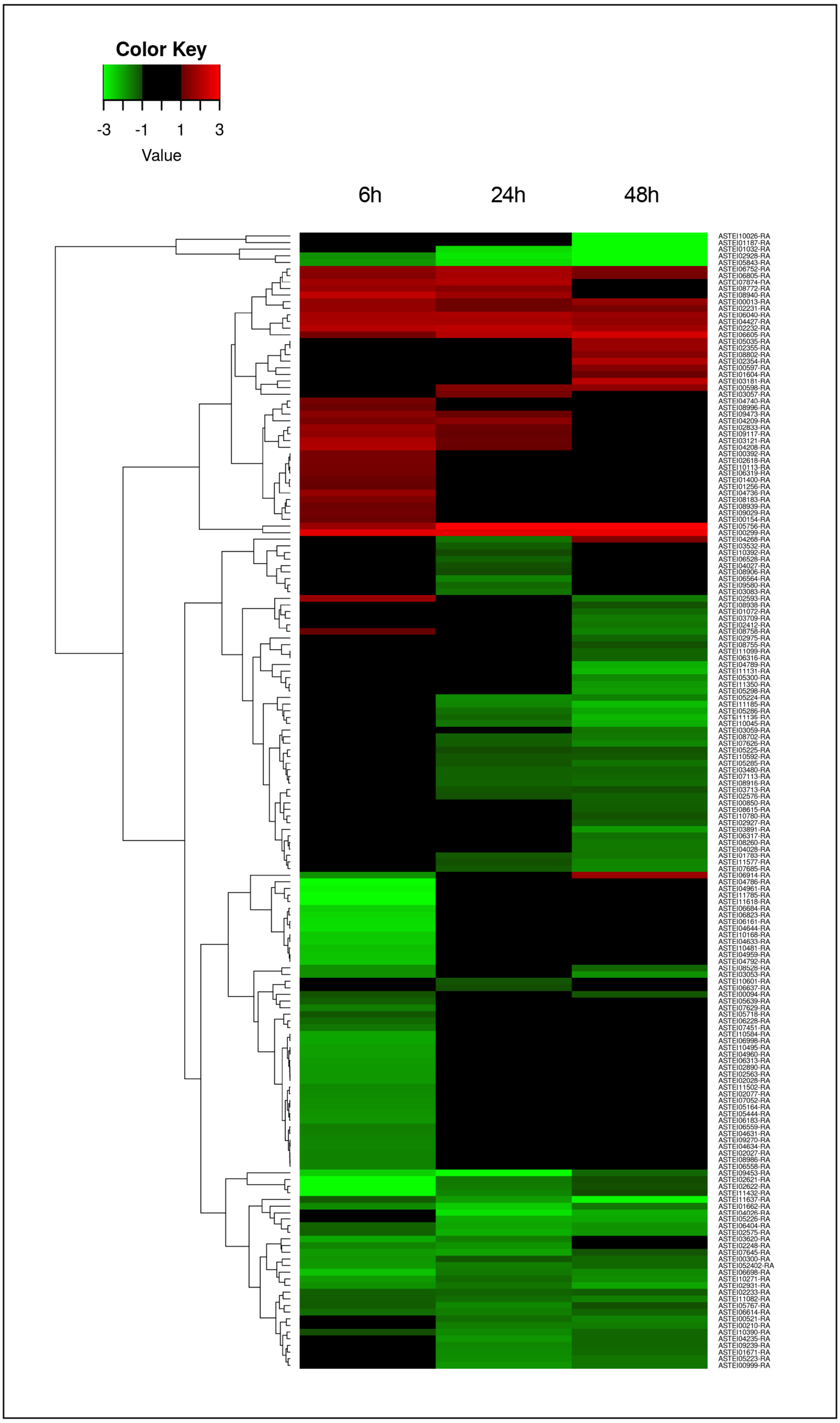

Figure S4. Cluster analysis of Cuticular Proteins. Clustering of genes encoding for Cuticular Proteins detected as differentially expressed at six, 24 and 48h of permethrin exposure. The Roman letters above the clusters refers to the main groups and several sub-groups as indicated in the Results section.

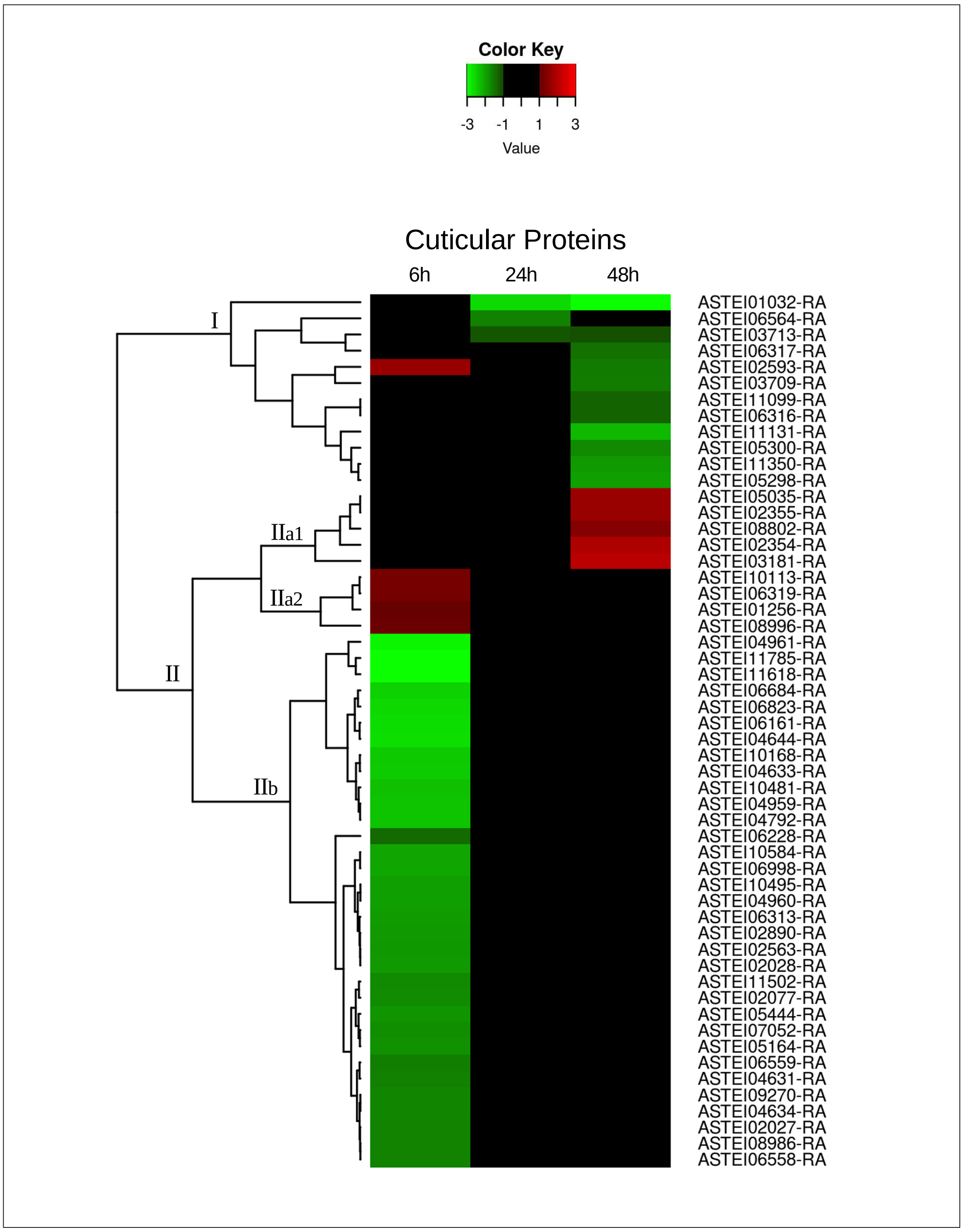

Figure S5. Validation of RNA-seq data by RT-qPCR. (A) RNA-Seq data. The expression value is expressed as log2FC. A gene was considered differentially expressed compared to untreated larvae if its adjusted p-value was less than 0.05 and its absolute log2 fold change was greater than 1. (B) RT-qPCR results. The expression level in untreated larvae was considered the basal level, which was set to 1. The internal reference gene rps7 for *Anopheles stephensi* was used to normalize expression levels. The values are expressed as means of three values and the bars show the standard deviations.

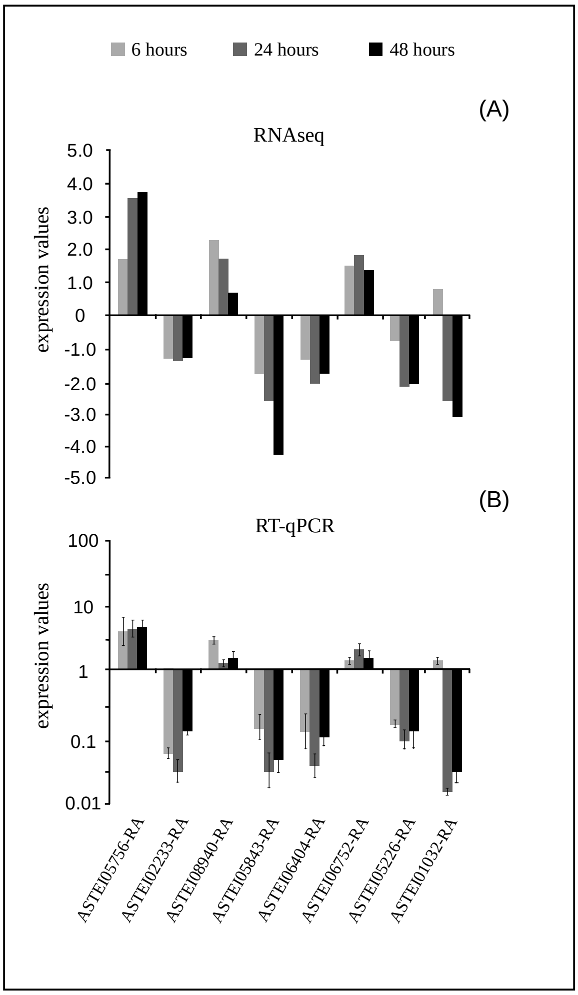

Supplement: Supplementary Information [file srep41312-s1.pdf]
